# Ultramicroporous Tröger's Base Framework Membranes With Ionized Sub‐nanochannels for Efficient Acid/Alkali Recovery

**DOI:** 10.1002/advs.202414280

**Published:** 2025-01-14

**Authors:** Haopan Sun, Ning Gan, Yuqing Lin, Baolong Wu, Yulong Qiu, Jingwen Su, Ziding Zhou, Fengyin Zou, Jianguo Yu, Hideto Matsuyama

**Affiliations:** ^1^ National Engineering Research Center for Comprehensive Utilization of Salt Lake Resources East China University of Science and Technology Shanghai 200237 P. R. China; ^2^ Research Center for Membrane and Film Technology Department of Chemical Science and Engineering Kobe University Kobe 6500034 Japan

**Keywords:** acid/alkali recovery, diffusion dialysis, ion‐exchange membrane, selective‐electrodialysis, Tröger's Base framework

## Abstract

Membrane technology holds significant potential for the recovery of acids and alkalis from industrial wastewater systems, with ion exchange membranes (IEMs) playing a crucial role in these applications. However, conventional IEMs are limited to separating only monovalent cations or anions, presenting a significant challenge in achieving concomitant H⁺/OH⁻ permselectivity for simultaneous acid and alkali recovery. To address this issue, the charged microporous polymer framework membranes are developed, featuring rigid Tröger's Base network chains constructed through a facile sol‐gel process. The intrinsic ultramicropore confinement and quaternary ammonium‐charged functional groups provide ultrahigh size‐sieving capability and enhanced Donnan exclusion for H⁺/OH⁻ selectivity; meanwhile, the internal protoplasmic channels of the polymer frameworks serve as highways for rapid ion transfer. The resulting membrane achieves high H⁺/Fe^2^⁺ and OH⁻/WO₄^2^⁻ selectivities of 694.4 and 181.0, respectively, for concurrent acid and alkali separation in diffusion dialysis and electrodialysis processes over extended operational periods (exceeding 1600 and 600 h, respectively), while maintaining remarkable transport rates. These results outperform most literature‐reported and nearly all commercially available membranes. This study validates the novel applicability of polymer framework materials with ionized angstrom‐scale channels and versatile functionalities in high‐performance IEMs for acid/alkali resource recovery.

## Graphical abstract

Charged microporous polymer framework membranes are developed featuring rigid Tröger's Base network structure. The intrinsic ultramicropore confinement and quaternary ammonium‐charged functional groups provide ultrahigh size‐sieving capability and enhanced Donnan exclusion for H⁺/OH⁻ selectivity; meanwhile, the internal protoplasmic channels of the polymer frameworks serve as highways for rapid ion transfer. The resulting QA‐TBF membrane achieves efficient acid and alkali separation in diffusion dialysis and electrodialysis processes.

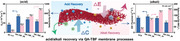

## 1 Introduction

Large amounts of waste acids and alkalis are produced in modern industries for various processes, such as acidic wastewater generated in iron and steel manufacturing, electroplating, and metal products while alkali wastewater is released in massive chlor‐alkali processing.^[^ ^1^, ^2^ ^]^ To minimize pollution and promote environmental conservation, there is an urgent demand for cost‐effective and efficient industrial wastewater recovery systems.^[^ ^3^ ^]^ Sustainable extraction of high‐purity acids, alkalis, or valuable metals from these effluents before they are discharged into the environment is crucial for reducing environmental impacts.^[^ ^4^ ^]^ Among the available separation approaches, dialysis‐driven technology is particularly attractive due to its operational simplicity, energy efficiency, and minimal chemical requirements.^[^ ^5^, ^6^ ^]^

The dialysis‐driven process utilizes an electric field or concentration gradient to transport ions from a concentrated solution through ion exchange membranes (IEMs) into a dilute solution.^[^ ^7^, ^8^ ^]^ Traditionally, IEMs are charge‐dense, allowing only the transport of ions with a charge opposite to that of the membrane (counterions) while excluding ions with the same charge (co‐ions).^[^ ^9^, ^10^ ^]^ Specifically, negatively charged membranes that permit cation passage are termed cation exchange membranes (CEMs), while positively charged membranes that allow anion passage are termed anion exchange membranes (AEMs).^[^ ^11^, ^12^ ^]^ The selective transport of counterions is one of the most desirable properties of CEMs and AEMs. Thus, engineering membranes capable of separating like‐charged species is a key prerequisite for extracting critical elements from complex systems using dialysis‐driven technology.^[^ ^13^ ^]^ To date, both CEMs and AEMs have been effectively employed in waste acid or alkali recovery processes. However, very few studies have been conducted on the development of membranes that can function efficiently in both processes, achieving the simultaneous rejection of multivalent anions and cations from highly complex industrial wastewater at the same time.^[^ ^14^, ^15^, ^16^ ^]^

Constructing ion transport channels is crucial for designing high‐performance IEMs. Inspired by the microstructure of the perfluorosulfonic acid membrane Nafion, traditional design strategies focus on microphase separation through the self‐assembly of charged polymers.^[^ ^17^, ^18^ ^]^ In these membranes, the continuous hydrophilic phase serves as the ion channel for H⁺/OH⁻ transport, while the hydrophobic phase provides dimensional stability.^[^ ^19^, ^20^ ^]^ Although such membranes are well‐developed and readily available, they are still constrained by the trade‐off between ionic conductivity and selectivity.^[^ ^21^ ^]^ This trade‐off arises from the microphase separation, where the hydrophilic regions undergo significant hydration‐induced swelling, leading to reduced ionic selectivity. To address this issue, microporous polymers, particularly solution‐processable polymers of intrinsic microporosity (PIMs), have garnered increasing attention as promising candidates for IEMs.^[^ ^22^, ^23^ ^]^ Instead of relying on dense hydrophilic regions like conventional IEMs, PIM membranes utilize intrinsic micropores, formed from the inefficient packing of rigid polymer chains, as twisted ion channels that combine size exclusion with high free‐volume‐induced permeability.^[^ ^24^ ^]^ These microporous polymer frameworks, with their uniform and durable angstrom‐scale pore channels, have proven highly effective as membrane separators in applications such as aqueous organic flow batteries, osmotic extraction devices, and high‐temperature fuel cells.^[^ ^25^ ^]^ Consequently, with optimized pore chemistry, PIM‐based IEMs show significant promise for acid/alkali recovery.

In this study, a novel series of functional polymer framework membranes was developed using rigid Tröger's Base framework (TBF) chains, transforming conventional IEMs into charged microporous membranes.^[^ ^26^ ^]^ The angstrom‐scale channels provided by the PIM network enhance ionic conductivity, enabling fast permeation of both H⁺ and OH⁻ via the Grotthuss mechanism.^[^ ^27^ ^]^ Meanwhile, the intrinsic micropores and charged quaternary ammonium groups endow the membrane with ultrahigh size‐sieving capability and significant Donnan exclusion toward multivalent ions with larger hydrated radii.^[^ ^28^ ^]^ As anticipated, the TBF membrane effectively differentiated H⁺ and OH⁻ from simulated industrial waste acid/alkali solutions under both concentration gradient‐driven and electric field‐driven dialysis processes, achieving high selectivities of 694.4 for H⁺/Fe^2^⁺ and 181.0 for OH⁻/WO₄^2^⁻, while maintaining remarkable transport rates for H⁺ and OH⁻. Notably, the resulting polymer membrane demonstrated operational stability for continuous acid/alkali recovery treatment over 1600 and 600 h, respectively. The performance observed in both acid/alkali recovery surpasses that of most reported membranes in the literature and nearly all commercially available membranes, paving the way for designing ionized microporous framework membranes for comprehensive resource extraction from industrial waste solutions.

## 2 Results and Discussion

### 2.1 Physico‐Chemical Characterizations of the QA‐TBF Membranes

**Figure 1:**
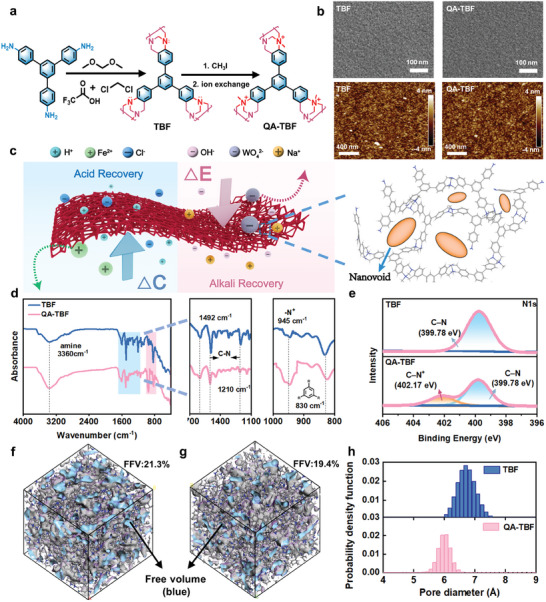
Syntheses and characterizations of Troger's Base framework membranes. a) Reaction route for positively charged TBF membranes (QA‐TBF). b) SEM images of the surface morphology and AFM images of TBF membrane and QA‐TBF membrane. c) Schematic showing the diffusion dialysis acid recovery (left) and electrodialysis alkali recovery (right) with QA‐TBF. The molecular model of the repeating unit illustrates the framework structure of QA‐TBF. d) FT‐IR spectra and detailed spectrogram of TBF (blue) and QA‐TBF (pink). e) N 1s core‐level spectra of TBF before and after quaternization treatment. f,g) Molecular dynamics simulation of free volume of TBF and QA‐TBF networks, respectively. h) Pore size distributions of TBF and QA‐TBF derived from PALS characterization.

The free‐standing TBF membranes were fabricated using an organic sol‐gel method with the TPB monomer, where the reactive amino sites readily polymerize to form a mechanically robust nanostructure with intrinsic microporosity.^[^ ^29^ ^]^ Additionally, a quaternization treatment was applied to introduce positively charged quaternary ammonium groups, resulting in the formation of QA‐TBF membranes. The content of these positively charged groups was adjusted by regulating the reaction times (**Figure** **1**a,c). Notably, the microporous structure provides a size‐sieving effect, allowing only small ions such as OH⁻ to pass through, while the positively charged groups create strong electrostatic exclusion, selectively blocking high‐valence metal ions and permitting only H⁺ to permeate. These versatile membranes, built upon Tröger's Base frameworks, show great potential for concurrent acid and alkali recovery applications.

The morphological microstructures and membrane thicknesses were examined using SEM and AFM, revealing surface‐dense and defect‐free configurations for both TBF and QA‐TBF membranes, with comparable roughness values ranging from 0.87 to 0.92 nm (Figure 1b). These results suggest that the introduced ammonium groups have a negligible impact on the structural integrity of the TBF membranes following surface quaternization. The thickness of the QA‐TBF membranes was easily controlled by adjusting the monomer concentrations in the casting solutions, resulting in thicknesses ranging from ≈60 to 80 µm in this study (Figures S1–S3, Supporting Information). The as‐obtained membranes were designated QA‐TBF_60, QA‐TBF_70, and QA‐TBF_80, corresponding to TPB concentrations of 0.04, 0.05, and 0.06 g mL^−1^, respectively. Additionally, the membrane thicknesses could be controlled by varying the amount of casting solution while maintaining a constant TPB monomer concentration of 0.06 g mL^−1^ (Figure S4, Supporting Information). The mechanical properties of the QA‐TBF membranes improved with increasing structural thickness (Figure S5, Supporting Information). Specifically, QA‐TBF_70 demonstrated a tensile strength of 16.1 MPa and an elongation rate of 18.6%, indicating that the PIM‐based microcellular framework membrane possesses sufficient mechanical robustness, attributed to its rigid framework chains.^[^ ^34^ ^]^

In contrast, a more pronounced change in surface chemistries could be observed before and after quaternization treatment. The scissoring characteristic peaks at 945, 830, and 3360 cm⁻¹, corresponding to the quaternary amine bond, trisubstituted benzene ring vibrations, and amine bands from TPB,^[^ ^30^ ^]^ respectively, were identified (Figure 1d). The QA‐TBF membrane exhibited a stronger XPS peak at 402.17 eV,^[^ ^31^ ^]^ corresponding to quaternary ammonium (−N⁺), indicating enhanced positive charges compared to the pristine TBF membrane, confirming a successful quaternization (Figures 1e and S7, Supporting Information). The effect of quaternization on the intrinsic microporosity of TBF and QA‐TBF membranes was evaluated using positron annihilation lifetime spectroscopy (PALS). The micropore sizes of both TBF and QA‐TBF membranes were primarily distributed within the ultramicropore range, with the QA‐TBF membranes exhibiting a slightly reduced pore width following quaternization (Figure 1h). Simulations of pore structures, based on polymer chain packing (Figures 1f,g), revealed that the gray/blue areas, representing larger fractional free volumes (FFV), were more prevalent in the pristine TBF membranes (21.3%) compared to QA‐TBF membranes (19.4%). Both experimental characterization (Figures 1 h and S8, Supporting Information) and simulations confirm that the QA‐TBF membrane possesses tighter structures with reduced microporosity, as the introduced bulky charged groups occupy the internal free volumes.

### 2.2 Ion‐Selective Transport of QA‐TBF Membranes

**Figure 2:**
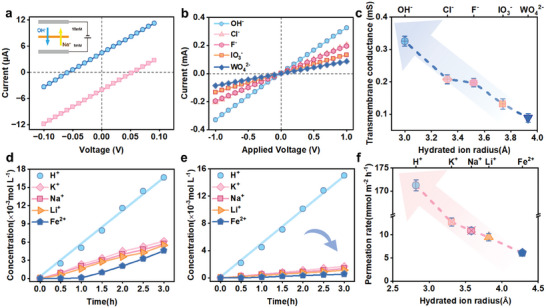
a) Current−voltage (*I*–*V*) curves of the QA‐TBF membrane under a ten fold concentration gradient in NaOH. b) *I*–*V* curves of the QA‐TBF membrane recorded in 10 mm sodium salt solutions with different anions. c) Transmembrane anion conductance of QA‐TBF membrane as a function of hydrated anion radius. d) The concentration of chloride solutions in the receiving chamber over time for selected cations, where the slopes indicate the permeability differences between TBF and e) QA‐TBF membranes for various cations. f) Cation permeation rates plotted against the hydrated cation radius through QA‐TBF membrane.

A two‐compartment diffusion cell was employed for current‐voltage (*I*–*V*) measurements to evaluate the membrane's selectivity for anions over cations. The *I*–*V* curves for a ten fold concentration gradient recorded the membrane potential of 46.1 mV. Using the Nernst equation, the transference numbers of the pristine QA‐TBF and TBF membranes were calculated to be 0.98 and 0.79 (**Figure** **2**a), respectively (Figure S9, Supporting Information), indicating superior anion/cation selectivity of the QA‐TBF membrane compared to the pristine TBF membrane. This enhanced selectivity can be attributed to the quaternary ammonium groups, which introduce positive charges within the rigid pore channels, repelling cations and promoting rapid anion transport. Furthermore, the separation capability of the QA‐TBF membrane for differentiating between anionic species was examined using various sodium salts (NaOH, NaCl, NaF, NaIO₃, and Na₂WO₄) under external potentials. The slopes of the I‐V curves reflect their transmembrane conductance (Figure 2b). The QA‐TBF membrane exhibited significantly higher currents for monovalent compared to divalent anions (Figure 2c), following the sequence OH⁻ > Cl⁻ > F⁻ > IO₃⁻ > WO₄^2^⁻, inversely related to their hydrated radii: OH⁻ (3.0 Å) < Cl⁻ (3.32 Å) < F⁻ (3.52 Å) < IO₃⁻ (3.74 Å) < WO₄^2^⁻ (3.93 Å).^[^ ^32^ ^]^ These results suggest that selective anion transport is primarily governed by the size‐sieving effect, with larger anions restricted by the angstrom‐scale channels, where the pore size (6 Å) closely matches the Stokes diameter of OH⁻ ions but is significantly smaller than that of WO₄^2^⁻.

The diffusion behaviors of cationic species through TBF‐based membranes were evaluated using concentration‐gradient‐driven dialysis. The feed‐side chambers were filled with chloride salt solutions (HCl, KCl, NaCl, LiCl, and FeCl₂), while deionized (DI) water was used in the permeate‐side chamber. The diffusivity of cations in QA‐TBF membranes was found to be lower than that in pristine TBF membranes, particularly for the divalent cation Fe^2^⁺, which exhibited a significant difference in permeation rates of 28.45 and 6.08 mmol m⁻^2^·h⁻¹ for TBF and QA‐TBF membranes, respectively (Figure 2d,e). This observation highlights the characteristic positive charge of the QA‐TBF membrane, which was corroborated via zeta potentials in Figure S10 (Supporting Information). However, both membranes exhibit comparably high H^+^ diffusion rates of 170 mmol m^−2^ h^−1^, indicating a neglected change of resistance toward the smallest H^+^ ion from the confined angstrom‐scale channels before and after quaternization. Furthermore, the permeation of cations across the QA‐TBF membrane followed the sequence H⁺ (2.82 Å) ≫ K⁺ (3.31 Å) > Na⁺ (3.58 Å) > Li⁺ (3.82 Å) > Fe^2^⁺ (4.28 Å),^[^ ^33^ ^]^ as shown in Figure 2f. Consequently, the combination of strong Donnan exclusion and size‐sieving effects allows the QA‐TBF membrane to effectively impede larger‐sized and higher‐valence cations, making it preferentially H⁺‐selective transport for acid recovery applications. Additionally, the membrane facilitates simultaneous selective transport of H⁺ and OH⁻ over other cations and anions, while maintaining continuous operation stability under acidic (0.5 m) and alkaline (0.5 m) conditions for 1600 and 600 h, respectively, with only minor performance degradation of 9.3% and 10.1% compared to their initial states (Figures S11 and S12, Supporting Information). The QA‐TBF membrane also demonstrates excellent mono/divalent ion selectivities, following the sequence of K⁺/Fe^2^⁺ (310.1) > Na⁺/Fe^2^⁺ (215.7) > Li⁺/Fe^2^⁺ (192.1), and Cl⁻/WO₄^2^⁻ (44.1) > F⁻/WO₄^2^⁻ (36.5) > IO₃⁻/WO₄^2^⁻ (23.9) over diffusion dialysis and electrodialysis processes under acid/alkali conditions (Figure S13, Supporting Information). The results highlight the significant potential of QA‐TBF membranes as effective separators for acid and alkali recovery applications. The results demonstrate that QA‐TBF holds significant potential for use as membrane separators for both alkali and acid recovery.

The ion exchange capacity (IEC) and hydration‐induced swelling properties of TBF‐based membranes were systematically investigated. The bulk water uptake of the pristine TBF and QA‐TBF membranes, which exhibited a high IEC of 1.76 mmol·g⁻¹ (Figure S14, Supporting Information), was determined by measuring the mass changes of the membranes under dry and hydrated conditions. A negligible difference in water uptake was observed between the pristine TBF (9.1%) and QA‐TBF membranes (14.9%), indicating minor swelling (4.3%) even after the introduction of quaternary ammonium groups (Figure S14, Supporting Information). Water contact angles of the membrane indicated an increase in hydrophilicity upon the introduction of quaternary amine groups, as evidenced by a decrease in surface contact angle from 77.5° to 66.5° (Figure S15, Supporting Information). The swelling ratio was plotted as a function of IEC (Figure S16, Supporting Information). Despite the significantly enhanced IEC, the QA‐TBF membrane displayed a relatively lower swelling ratio, comparable to the latest‐reported PIMs membranes and superior to the commercial Nafion membrane and other conventional microporous membranes. This performance can be attributed to the rigid micropore confinement characteristic of the Tröger's Base framework,^[^ ^34^ ^]^ in contrast to the more flexible amide linkages present in traditional polymeric IEMs.

### 2.3 Selective‐Electrodialysis of Alkali Recovery for QA‐TBF Membranes

**Figure 3:**
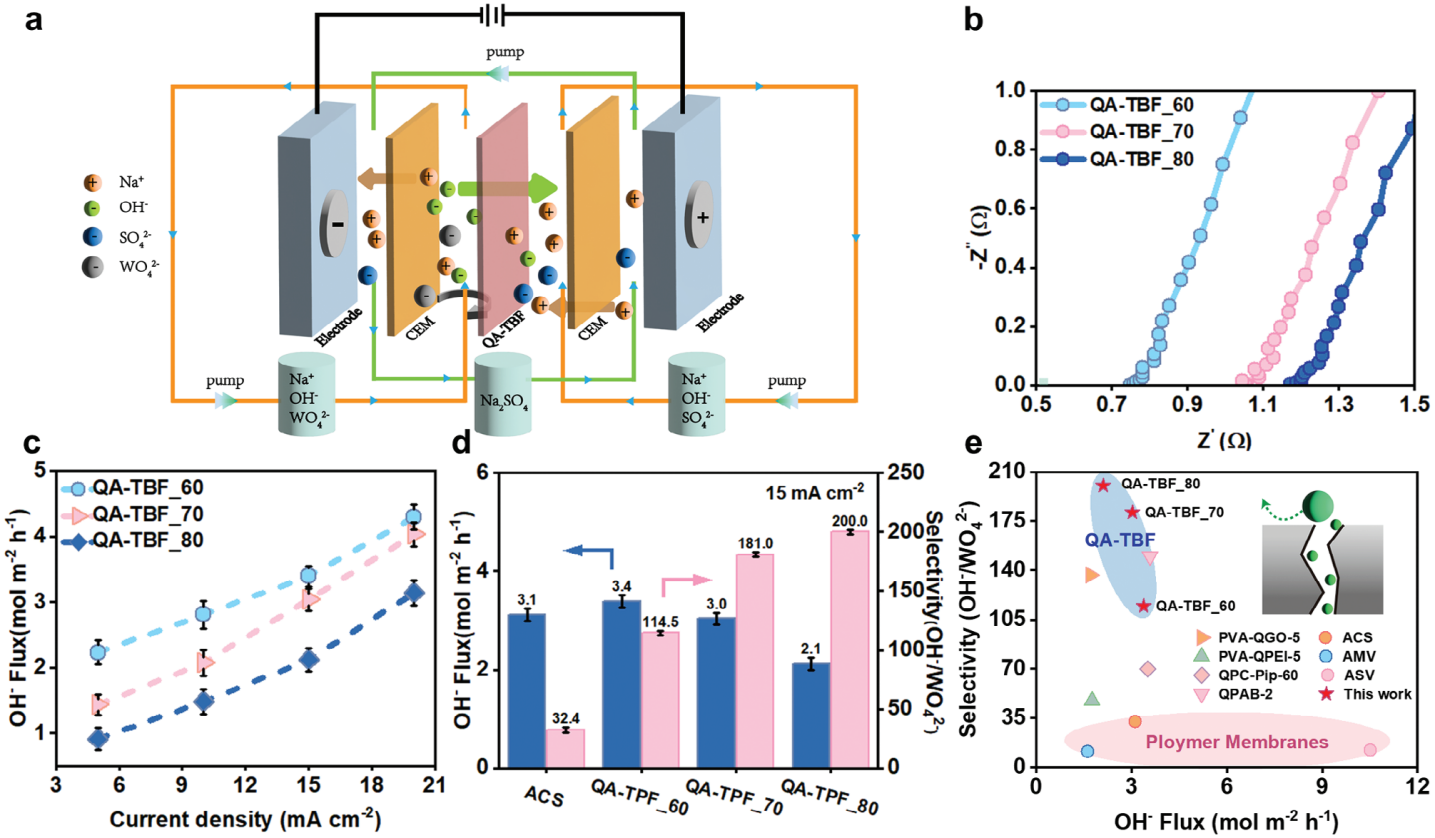
a) Schematic diagram of the selective‐electrodialysis setup for alkali recovery. b) EIS spectra of QA‐TBF membranes with varying thicknesses in 0.5 m NaOH. c) OH^−^ flux of QA‐TBF membranes as a function of current density for different membrane thicknesses. d) Comparison of OH^−^ flux and OH^−^/WO_4_^2−^ selectivity between the commercial ACS and QA‐TBF membranes with varying membrane thickness at 15 mA·cm^−2^. e) Comparison of OH^−^ flux and OH^−^/WO_4_^2−^ selectivity of QA‐TBF membranes in selective‐electrodialysis with representative membranes from the literature. Detailed values are provided in Table S2 (Supporting Information).

A selective‐electrodialysis cell equipped with QA‐TBF membranes was employed for the recovery of OH‐ from a NaOH and Na₂WO₄ solution mixture, simulating a typical industrial waste alkali system (**Figure** **3**a). Driven by an electric field, OH⁻ and WO₄^2^⁻ ions compete to permeate through the QA‐TBF membrane, and then become enriched in the concentrated compartment. This process relies on the selective transport of OH⁻ over WO₄^2^⁻. The selective‐electrodialysis was conducted under current densities ranging from 5 to 20 mA·cm^−^^2^, below the limiting current density of the membranes (Figure S17, Supporting Information) to prevent water electrolysis, which would otherwise increase energy consumption and reduce current efficiency.^[^ ^35^ ^]^ The OH⁻ ion transport resistance of the QA‐TBF membranes in 0.5 m NaOH solutions was measured using electrochemical impedance spectroscopy (EIS) (Figure 3b). Compared to QA‐TBF_60, thicker membranes exhibited progressively higher ohmic resistance, calculated at 1.32, 1.85, and 2.08 Ω·cm^2^ for QA‐TBF_60, QA‐TBF_70 and QA‐TBF_80, respectively, by subtracting the filmless resistance from the total ohmic resistance of the cell. A similar trend was observed for QA‐TBF membranes prepared using different casting solution volumes (Figure S18, Supporting Information). Despite this increase in resistance, the newly developed QA‐TBF membranes maintained high OH⁻ ion conductivity, ranging from 0.30 to 0.36 mS, comparable to the commercial ASTOM membrane (0.35 mS) under the same testing condition (Figure S19, Supporting Information). Additionally, OH⁻ ion flux increased with rising current density due to the stronger electric field‐driven propulsion (Figure 3c), consistent with the ion‐selective transport behavior observed in AEMs in previous studies.

A series of electrodialysis experiments were conducted at current densities from 5 to 20 mA·cm⁻^2^ using QA‐TBF membranes of varying thicknesses. As the membrane thickness increased from 60 to 80 µm, the OH⁻/WO₄^2^⁻ selectivity significantly increased from 114.5 to 200, achieving a nearly two fold enhancement. However, the OH⁻ flux experienced a slight decline compared to WO₄^2^⁻, with a modest reduction from 3.4 to 2.1 mol·m⁻^2^·h⁻¹ at 15 mA·cm⁻^2^ (Figure 3d). Similar trends were observed at current densities of 10 and 20 mA·cm⁻^2^ (Figures S20 and S21, Supporting Information), where OH⁻ flux changed slightly across all QA‐TBF membranes, while OH⁻/WO₄^2^⁻ selectivity showed more pronounced increases. This suggests that the mobility of the larger WO₄^2^⁻ ions (3.93 Å) was effectively hindered by the expanded and tortuous monovalent ion‐selective transport pathway, while the smaller OH⁻ ions (3.0 Å), being significantly smaller than the micropores of the QA‐TBF membranes (0.6–0.7 nm), could permeate easily. This is because to maintain charge balance during electrodialysis at a given current density, ion transport must accompany the movement of electron carriers.^[^ ^36^ ^]^ Therefore, considering the larger steric hindrance confronted by WO₄^2^⁻ ions in partitioning into the membrane pore channels, accelerated OH⁻ transport becomes the dominant ion flux pathway. Compared with state‐of‐the‐art GO‐based,^[S23]^ polymer‐based,^[S24]^ and commercially available membranes,^[S25]^ our newly‐developed QA‐TBF_70 membrane, with a thickness of 70 µm, demonstrates excellent alkali recovery capabilities, achieving higher OH^−^ flux and competitive OH^−^/WO_4_^2−^ selectivity (Figure 3e, summarized in Table S2, Supporting Information). This superior performance can be attributed to the intrinsic microporosity and rigid confinement provided by the Tröger's Base frameworks,^[^ ^29^ ^]^ enabling rapid OH^−^ ion‐selective transport.

### 2.4 Diffusion Dialysis of Acid Recovery for QA‐TBF Membranes

**Figure 4:**
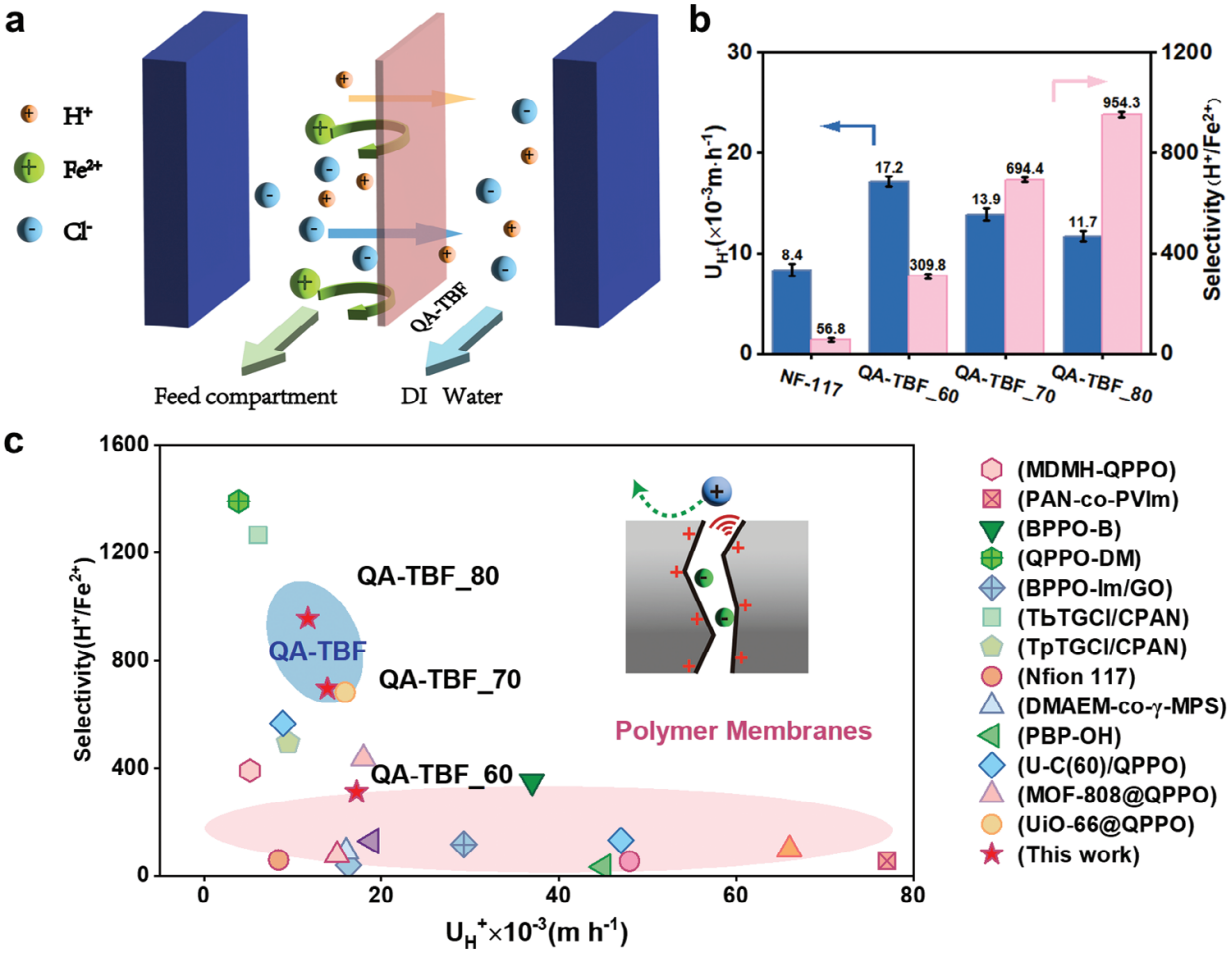
a) Schematic diagram of the diffusion dialysis setup for acid recovery. b) Comparison of H^+^ flux (U_H_^+^) and H^+^/Fe^2+^ selectivity between the commercial Nafion‐117 and QA‐TBF membranes with varying membrane thicknesses. c) Comparison of U_H_^+^ and H^+^/ Fe^2+^ selectivity of QA‐TBF membranes during acid recovery via diffusion dialysis, with representative membranes from the literature. Detailed values are provided in Table S3 (Supporting Information).

The H⁺ ion‐selective transport properties of both TBF‐based and commercial Nafion membranes were examined through concentration‐gradient‐driven diffusion measurements (**Figure** **4**a) to evaluate their acid recovery performance from an H⁺/Fe^2^⁺ mixture solution, simulating typical industrial waste acid systems.^[^ ^37^ ^]^ The acid recovery efficiency was assessed based on the H⁺ diffusion rate and H⁺/Fe^2^⁺ selectivity, which ranged from 17.2 to 11.7 mm·h⁻¹ and 309.8 to 954.3, respectively, as the membrane thickness increased from 60 µm (QA‐TBF_60) to 80 µm (QA‐TBF_80) (Figure 4b). These trends aligned with the observations from the electrically driven permeation experiments (Figures 3d and S20, and S21, Supporting Information). Figure 4b further illustrates that, despite Nafion membranes being widely used as cation exchange membranes, their H⁺/Fe^2^⁺ selectivity is relatively low, with a value of only 56.8, which significantly hampers their practical application in processes requiring efficient ion selectivity. This limitation is similarly observed in all‐vanadium redox flow batteries (VRFB),^[^ ^38^ ^]^ where Nafion membranes demonstrate high H⁺ conductivity but suffer from substantial VO^2^⁺ ion crossover, leading to self‐discharge and reduced operational lifespan.^[^ ^39^ ^]^ In contrast, the QA‐TBF_70 membrane exhibited a significantly higher H⁺/Fe^2^⁺ selectivity of 694.4, nearly 12‐fold greater than that of Nafion, while maintaining an H⁺ diffusion rate of 13.9 mm·h⁻¹, which was notably higher than the 8.4 mm·h⁻¹ recorded for Nafion. These results underscore the potential of QA‐TBF membranes for high‐efficiency H⁺ transport and superior acid recovery in industrial applications.

Notably, the performance of our newly developed QA‐TBF_70 membrane surpasses that of recently reported ion‐sieving membranes, including conventional PFSA, GO‐based,^[S33]^ MOF/COF‐based,^[S35]^ polyamide,^[S36]^ and other commercially available membranes (Figure 4c, summarized in Table S3, Supporting Information). The exceptional selectivity of the QA‐TBF membranes can be ascribed to the significant permeation resistance experienced by Fe^2^⁺ within the ionized angstrom‐scale channels. This resistance, arising from both structural steric hindrance and Donnan exclusion, is significantly greater than that encountered by H⁺ ions (Figures S18 and S22, Supporting Information). During diffusion dialysis, Cl⁻ ions migrate much faster across the QA‐TBF membrane than cations, leading to a high membrane potential and an impressive transference number of 0.99 in 1/10 mm KCl solutions (Figure S23, Supporting Information). As a result, more H⁺ ions, featuring smaller and monovalent ones, pass rapidly through the membrane with minimal mass transfer resistance to maintain charge neutrality in the permeate‐side chamber.^[^ ^40^ ^]^ In contrast, larger and multivalent Fe^2^⁺ ions are retained due to the significantly enhanced permeation resistance, driven by the synergistic effects of size sieving and electrostatic exclusion within the angstrom‐scale channels of QA‐TBF membranes.

## 3 Conclusion & Perspective

In summary, we successfully developed a Tröger's Base polymer framework membrane featuring intrinsic ultramicroporosity and charged quaternary ammonium groups. The micropores, formed by the inefficient packing of rigid polymer chains, function as twisted ion channels, providing favorable permeability and excellent size‐sieving properties where larger anions are restricted by the angstrom‐scale channels. Furthermore, while H⁺ ions encounter minimal resistance entering these confined angstrom‐scale channels, higher‐valence cations experience a pronounced Donnan exclusion effect. As such, the resulting QA‐TBF membrane concomitantly differentiated H⁺ and OH⁻ from simulated industrial waste acid and alkali solutions in both diffusion dialysis and electrodialysis processes, achieving high selectivities of 694.4 for H⁺/Fe^2^⁺ and 181.0 for OH⁻/WO₄^2^⁻ at the same time, while maintaining remarkable transport rates. The optimal QA‐TBF_70 membrane, with a thickness of 73 µm, outperforms both reported and commercial membranes by balancing the trade‐off between permeability and selectivity for H^+^ and OH^−^ ions. More importantly, it demonstrates excellent long‐term operation stability in both acidic/alkaline environments over 1600 and 600 h. Despite the successful simultaneous acid/alkali separation, different functional substituent groups within Tröger's Base frameworks significantly influence membrane performance. For instance, bulky substituents with strong hydrophobicity or flexible long chains may reduce the free volume, resulting in structural changes that can simultaneously affect both membrane ionic conductivity and operational stability. Therefore, future research should focus on investigating the structural effects of substituent groups on membrane performance to better understand and optimize the effectiveness of PIM‐based membranes for diversified separation applications.

## Conflict of Interest

The authors declare no conflict of interest.

## Supporting information



Supporting Information (Dear editor, the authorship of this SI is incorrect. Please kindly refer to and use the newly attached one)

## Data Availability

The data that support the findings of this study are available in the Supporting Information of this article.
