# Supplementary material for: Ultramicroporous Tröger's Base Framework Membranes With Ionized Sub‐nanochannels for Efficient Acid/Alkali Recovery
Source: Adv Sci (Weinh). 2025 Jan 14;12(9):2414280. doi: 10.1002/advs.202414280 (PMC11884555; doi:10.1002/advs.202414280)
Supplement: Supplementary file 1 — Supporting Information (Dear editor, the authorship of this SI is incorrect. Please kindly refer to and use the newly attached one) [file ADVS-12-2414280-s001.docx]

**Supporting Information**

**Ultramicroporous Tröger’s Base Framework Membranes with Ionized Sub-nanochannels for Efficient Acid/Alkali Recovery**

Haopan Sun, Ning Gan, Yuqing Lin, Baolong Wu, Yulong Qiu, Jingwen Su, Ziding Zhou,

Fengyin Zou, Jianguo Yu, and Hideto Matsuyama

[*] H, Sun, Y. Lin, N. Gan, B. Wu, Y. Qiu, J. Su, Z. Zhou, F. Zou, J. Yu
National Engineering Research Center for Comprehensive Utilization of Salt Lake Resources
East China University of Science and Technology
Shanghai 200237, P. R. China.

E-mail: [linyuqing@ecust.edu.cn](mailto:linyuqing@ecust.edu.cn)

H. Matsuyama

Research Center for Membrane and Film Technology, Department of Chemical Science and Engineering

Kobe University

Kobe 6500034, Japan

**Table of contents**

[**Materials** 5](#_Toc185099675)

[**Experimental Procedures** 5](#_Toc185099676)

[1. Preparation of the QA-TBF membranes 5](#_Toc185099677)

[2. Characterizations 6](#_Toc185099678)

[3. Selective-ion transport 7](#_Toc185099679)

[4. Selective-electrodialysis of alkali recovery 8](#_Toc185099680)

[5. Diffusion dialysis of acid recovery 9](#_Toc185099681)

[6. Supplementary membrane property measurements 10](#_Toc185099682)

[6.1 Water uptake and swelling ratio 10](#_Toc185099683)

[6.2 Ion exchange capacity 11](#_Toc185099684)

[6.3 Electrochemical impedance spectroscopy (EIS) 11](#_Toc185099685)

[6.4 Zeta potential 12](#_Toc185099686)

[6.5 Limiting current density 12](#_Toc185099687)

[6.6 Simulations of free volume 13](#_Toc185099688)

[**Supplementary figures and data** 14](#_Toc185099689)

[Figure S1. Digital photo of the TBF membrane (a) and QA-TBF membrane (b). 14](#_Toc185099690)

[Figure S2. SEM images illustrating the surface morphology of (a) TBF, (b) QA-TBF_60, (c) QA-TBF_70, and (d) QA-TBF_80. 15](#_Toc185099691)

[Figure S3. Cross-sectional SEM images of (a) TBF, (b) QA-TBF_60, (c) QA-TBF_70, and (d) QA-TBF_80, revealing the internal structural features and layer compositions of each sample. 16](#_Toc185099692)

[Figure S4. Cross-sectional SEM images of (a) QA-TBF_70, (b) QA-TBF_80, and (c) QA-TBF_90 fabricated by varying the amount of casting solution at 3.2, 4.0, and 4.8 mL. 17](#_Toc185099693)

[Figure S5. Stress-strain curves of QA-TBF membranes. 18](#_Toc185099694)

[Figure S6. The three-dimensional structure of QA-TBF network. 19](#_Toc185099695)

[Figure S7. (a) Full XPS spectra of TBF and QA-TBF, (b) High-resolution XPS spectra of C1s for TBF and QA-TBF. 20](#_Toc185099696)

[Figure S8. (a) CO_2_ sorption isotherms of TBF and QA-TBF at 273 K. (b) Pore size distributions of TBF and QA-TBF derived from CO_2_ sorption. 21](#_Toc185099697)

[Figure S9. Current−voltage (I−V) curves of the TBF membrane under a 10-fold concentration gradient in NaOH. 22](#_Toc185099698)

[Figure S10. Zeta Potential of TBF and QA-TBF membrane at different pH values. 23](#_Toc185099699)

[Figure S11. (a) Selectrodialysis alkali recovery for long cycle testing. (b) Diffusion dialysis acid recovery for long cycle testing. 24](#_Toc185099700)

[Figure S12. SEM images illustrating the surface morphology of (a) pristine QA-TBF_70, (b) QA-TBF_70 after soaking in 1M HCl solution for two weeks, (c) QA-TBF_70 after soaking in 1M NaOH solution for two weeks. (d) FT-IR spectra and detailed spectrogram of QA-TBF_70 in three states. (e) N 1s core-level spectra of QA-TBF_70 in three states, the upper part represents pristine, the middle represents acid treatment and the bottom represents alkali treatment. 25](#_Toc185099701)

[Figure S13. Salinity concentration-driven ion-selective transporting behavior and structural characterization analysis of QA-TBF. (a) Crossover diffusion behavior and monovalent (X^-^)/multivalent (WO_4_^2-^) ion selectivity in 0.2 M NaOH solution. (b) Crossover diffusion behavior and monovalent (M^+^)/multivalent (Fe^2+^) ion selectivity in 0.2 M HCl solution. 26](#_Toc185099702)

[Figure S14. (a) Water uptake and swelling ratio of TBF and QA-TBF membranes as a function of ion exchange capacity (IEC). (b) N 1s core-level spectra of TBF with different degrees of quaternization. 27](#_Toc185099703)

[Figure S15. Contact angle of TBF and QA-TBF membranes. 28](#_Toc185099704)

[Figure S16. Swelling ratio as a function of ion exchange capacity (IEC) for QA-TBF and other reported membranes, with detailed values presented in Table S1 for reference. 29](#_Toc185099705)

[Figure S17. Current-voltage (I-V) curves of QA-TBF membranes with varying thicknesses. 30](#_Toc185099706)

[Figure S18. EIS spectra of QA-TBF membranes fabricated using different amounts of casting solutions in (a) 0.5 M HCl and (b) 0.5 M NaOH. 31](#_Toc185099707)

[Figure S19. Transmembrane anion conductance (OH⁻ and WO₄²⁻) plotted against the ACS (ASTOM) and QA-TBF membranes with varying thicknesses. 32](#_Toc185099708)

[Figure S20. OH⁻ flux and OH⁻/WO₄²⁻ selectivity of ACS (ASTOM) and QA-TBF membranes with varying thicknesses, measured at a current density of 10 mA cm⁻². 33](#_Toc185099709)

[Figure S21. OH⁻ flux and OH⁻/WO₄²⁻ selectivity of ACS (ASTOM) and QA-TBF membranes with varying thicknesses, measured at a current density of 20 mA cm⁻². 34](#_Toc185099710)

[Figure S22. EIS spectra of QA-TBF membranes with varying thicknesses in (a) 0.5 M HCl and (b) 0.5 M FeCl₂. 35](#_Toc185099711)

[Figure S23. Current−voltage (I−V) curves of QA-TBF membranes subjected to a 10-fold concentration gradient of KCl. 36](#_Toc185099712)

[**Supplementary Tables** 37](#_Toc185099713)

[Table S1. Swelling ratio (SR) as a function of IEC value for membranes derived from polymer frameworks, PIMs, and the commercial Nafion membrane. 37](#_Toc185099714)

[Table S2. Summary of membranes used in the electrodialysis-based alkali recovery. 38](#_Toc185099715)

[Table S3. Summary of membranes used in the diffusion dialysis-based acid recovery. 39](#_Toc185099716)

[**Reference** 40](#_Toc185099717)

# Materials

1,3,5-Tris(4-aminophenyl) benzene (TPB), trifluoroacetic acid (TFA) Dimethoxymethane (DMM) and methyl iodide were purchased from TCI (Shanghai) Development Co., Ltd. Ferrous chloride tetrahydrate (FeCl_2_·4H_2_O), sodium tungstate dihydrate (Na_2_WO_4_·2H_2_O), hydrochloric acid (HCl) and sodium hydroxide (NaOH) were acquired from Sinopharm Chemical Reagent Co. Ltd. and used without additional purification. Inorganic salt sodium chloride (NaCl), sodium fluoride (NaF), sodium iodate (NaIO_3_), potassium chloride (KCl) and lithium chloride (LiCl) were purchased from Meryer (Shanghai) Biochemical Technology Co., Ltd. Deionized water was used throughout this study. The ACS membranes used in selective electrodialysis were obtained from ASTOM Corporation (Japan). The Nafion 117 membranes used in diffusion dialysis were obtained from Chemours Chemical (Shanghai) Co., Ltd.

# Experimental Procedures

## 1. Preparation of the QA-TBF membranes

Tröger's Base framework (TBF) membranes were synthesized via a sol-gel method.^[1]^ TPB (0.140 g) was mixed with DMM (0.181 g) in a round-bottomed flask at 0 °C. TFA (1.4 mL) was gradually added to the mixture, which was stirred at 0 °C for 30 minutes before being allowed to reach room temperature (25 °C) until a viscous solution formed. The as-obtained viscous solution was diluted with additional TFA (2.0 mL) and poured onto a flat glassware, followed by a heat treatment at 30 °C for 3 hours and then transferred to 40 °C until completely dried. After that, the dried membranes were immersed in 0.1 M KOH aqueous solution to eliminate excess TFA, with membrane thickness controllable through concentration adjustments prior to casting.

The resulting TBF membranes were further immersed in a 40 wt % mixture of methanol and methyl iodide for 6 hours to initiate functional quaternization, with the degree of quaternization adjustable by varying the reaction time. Following this, the quaternized TBF (QA-TBF) membranes were immersed in a 1 M KCl aqueous solution for 24 hours for anion exchange, and then washed with deionized water to remove any residual salts. The degree of quaternization increases with reaction time.

Additionally, to address potential defect formation during membrane fabrication, particularly concerning scalability, maintaining the homogeneity of membrane composition is critical. During upscaling, ensuring consistent mixing of precursor materials and functionalization agents becomes more challenging, which may lead to batch-to-batch variations. At larger scales, achieving uniform membrane thickness can also be difficult, particularly when casting onto large support substrates, potentially resulting in performance inconsistencies such as variations in permeability and selectivity. Consequently, scaling the fabrication process will require more sophisticated control over key parameters, including reactor shaking frequency, reaction temperature, and reactive time, to ensure consistent membrane quality and performance.

## 2. Characterizations

^13^C solid-state nuclear magnetic resonance (NMR) was conducted on Bruker AVANCE III 400 WB. Attenuated Total Reflection Infrared-Fourier transform infrared reflection (ATR FTIR) characterization (Thermo Fisher Scientific Nicolet iS20) and X-ray Photoelectron Spectroscopy (XPS) characterization (Thermo Scientific K-Alpha) were conducted to investigate the molecular structure and chemical composition of TBF membranes. The water contact angle was measured by an angle meter (SL200B, Solon Tech Co., ltd, China). Scanning electron microscope (SEM) characterization (ZEISS Gemini SEM 300) and atomic force microscopy (AFM) characterization (Bruker Scanasyst Dimension Icon AFM, using a peak force tapping mode in ambient air) were conducted to investigate the surface microstructure. Brunauer-Emmett-Teller (BET) measurements (ASAP 2640, Micromeritics 3Flex, USA) and Positron Annihilation Technique (PAT) measurements (DPLS3000) were conducted to investigate the surface area and pore distribution of TBF membranes. The surface charging properties of the TBF and QA-TBF were examined using an electrokinetic analyzer (SurPass 3, Anton Paar, Australia). Inductively coupled plasma (ICP) characterization (ICP-OES, ARCOS, SPECTRO, Germany) was conducted to determine the concentration of trace anions and cations.

## 3. Selective-ion transport

The anion-selective transport of ions with varying sizes across the TBF membrane was evaluated using current-voltage (I-V) measurements in a two-compartment H-cell. The compartments were separated with the TBF membrane (surface area: 1.8 cm^2^) and filled with 30 mL of 0.01 mol L^-1^ NaX (X represents OH^-^, Cl^-^, F^-^, IO_3_^-^, or WO_4_^2-^, respectively) electrolyte solution. Prior to measurement, the TBF membranes were pre-immersed in the corresponding electrolyte solution for 4 hours to achieve adequate equilibrium. The salt-bridged Ag/AgCl electrode was placed in each compartment and connected to an Autolab PGSTAT302N (Metrohm AG), which supplied a direct voltage that swept from -1 to 1 V at a scan rate of 0.01 V s^-1^. The resulting electrical signals were processed using NOVA2 (Metrohm AG) to plot I-V curves. The transmembrane conductance was derived by fitting the slope of the I–V curve with linear least squares regression, while the solution resistance was neglected. Similarly, the voltage was swept from -0.1 V to 0.1 V, with both chambers filled with a 10-fold concentration gradient of NaOH solution (0.001 M and 0.01 M). The membrane potential was determined by the x-axis intercept of the I-V curve, and transference number, t- was calculated using Equations ^[2]^:

$t_{-}=\frac{1}{2}(\frac{E_{m}}{\frac{RT}{zF}\ln(\frac{\gamma_{H}C_{H}}{\gamma_{L}C_{L}})})$

where R is the gas constant, T is the temperature of the solution, F is the Faraday constant, and z is the ionic valence state. γ, C and E_m_ are ion activity coefficients, ion concentration, and membrane potential respectively. t- is the number of anionic migrations number.

The permeation rate of cations across the TBF membrane under concentration gradients was measured using a diffusion H-cell, wherein the two compartments of the diffusion H-cell were separated by a TBF membrane. The feed-side compartment was filled with 60 mL of 0.2 mol L^-1^ XCl (X represents H^+^, K^+^, Na^+^, Li^+^, or Fe^2+^, respectively) electrolyte solution, alternatively, 0.2 mol L^-1^ mixed ion solution (K^+^, Na^+^, Li^+^ and Fe^2+^) under acidic condition or 0.2 mol L^-1^ mixed ion solution (Cl^-^, F^-^, IO_3_^-^ and WO_4_^2-^) under alkaline condition, while the permeate-side compartment was filled with 60 mL DI water. Stirring in both compartments was conducted vigorously to minimize concentration polarization. The ion concentration was calculated according to pre-established concentration-conductivity standard curves, with conductivity measured using a conductivity meter (LAQUAtwin EC-22 from HORIBA Advanced Techno, Co., Ltd.). Three sets of each test were performed, and the ion diffusion coefficient (P) and permeation rate (J) were calculated using Equations (2) and (3) according to Fick's law ^[3]^:

$P=\frac{dc_{t}}{dt}\frac{Vd}{A(C_{0}-C_{t})}$

$J=-P\frac{\Delta C}{d}=-P\frac{(C_{t}-C_{0})}{d}$

where V is the volume of the solution in the compartments (60 mL), d is the thickness of the membrane, A is the effective membrane area (1.8 cm^2^), t is the experiment time (3 h), C_0_ is the initial solute concentration in the feed-side compartment (0.2 mol L^-1^), and C_t_ is the solute concentration in the permeate-side compartment.

## 4. Selective-electrodialysis of alkali recovery

The capabilities of the AEMs including TBF and ACS membranes for alkali recovery were determined by measuring the selective transport of OH^-^ over WO_4_^2-^ in the selective-electrodialysis process. The electrodialysis cell consists of four compartments, including a concentrated compartment, a diluted compartment, and two electrode compartments. Two titanium electrodes, coated with ruthenium, were used for the anode and cathode, respectively. The diluted compartment was filled with a 200 mL binary mixture solution with both NaOH and Na_2_WO_4_ concentrations of 0.5 mol·L^-1^; meanwhile, the concentrated compartment was filled with 200 mL of 0.1 mol·L^-1^ Na_2_SO_4_. The other electrode compartments were filled with 200 mL of 0.3 mol·L^-1^ Na_2_SO_4_. The TPB membranes, with an effective membrane area of 7.0 cm^2^, were sandwiched between the diluted and concentrated chambers, where the corresponding solutions were circulated inside each compartment at a flow rate of 30 mL·min^-1^. Before measurement, the TBF membranes were equilibrated for 10 min in the electrodialysis cell. Subsequently, a constant current with densities ranging from 5~20 mA·cm^-2^ was applied and maintained for 1 hour. The OH^-^ concentration in the concentrated compartment was titrated, whereas the WO_4_^2-^ concentration was determined using ICP.

Three sets of each test were performed, and the OH^-^ and WO_4_^2-^ fluxes across the TBF-TPB membrane were calculated using Equations ^[4]^:

$J_{OH^{-}}=\frac{(C_{OH^{-}}-C_{OH^{-}}^{0})V}{At}$

$J_{WO_{4}^{2-}}=\frac{(C_{WO_{4}^{2-}}-C_{WO_{4}^{2-}}^{0})V}{At}$

where $J_{OH^{-}}$ and $J_{WO_{4}^{2-}}$ are the OH^-^ and WO_4_^2-^ fluxes during electrodialysis process, respectively. $C_{OH^{-}}^{0}$ and $C_{WO_{4}^{2-}}^{0}$are the initial OH^-^ and WO_4_^2-^ concentrations, while $C_{OH^{-}}$ and $C_{WO_{4}^{2-}}$ are their final concentrations in the concentrated compartment before and after the electrodialysis, respectively. V is the volume of solution in the concentrated cell (mL); A is the effective area of the membrane (cm^2^); t is the experiment time (h), respectively.

The OH^-^/ WO_4_^2-^ selectivity (S) of the TBF membrane could be calculated using Equation ^[5]^:

$S=\frac{J_{OH^{-}}C_{dWO_{4}^{2-}}}{J_{WO_{4}^{2-}}C_{dOH^{-}}}$

where $C_{dOH^{-}}$ and $C_{dWO_{4}^{2-}}$ are the initial concentrations of ions in the diluted compartment, respectively.

## 5. Diffusion dialysis of acid recovery

The capabilities of the AEMs, including TBF and Nafion 117 membranes, for acid recovery were determined by measuring the selective transport of H^+^ over Fe^2+^ via diffusion dialysis, in which the applied cell comprises two compartments. The TBF membrane, with an effective membrane area of 1.8 cm^2^, was sandwiched between the feed-side and permeate-side chambers. Prior to measurement, the membrane was equilibrated for 24 hours in an HCl solution, followed by washing with DI water. The feed-side compartment was filled with 60 mL of mixture solution containing 0.35 mol·L^-1^ HCl and 0.2 mol·L^-1^ FeCl_2_; while the permeate-side compartment was filled with 60 mL of DI water. Stirring was conducted with a magnetic stirrer in each compartment to minimize concentration polarization, and the diffusion dialysis was performed for 1 hour at room temperature. The H^+^ concentration in the permeate-side compartment was titrated and the Fe^2+^ concentration was determined using ICP.

Three sets of each test were performed, and the H^+^ and Fe^2+^ dialysis coefficients were calculated using Equation ^[6]^:

$U=\frac{M}{At\Delta C}$

where M is the amount of a component in moles transported, A is the effective area of membrane (1.8 m^2^), t is the experiment time (3 h), and ΔC is the average logarithmic concentration (mol·L^-1^) between the two compartments and can be calculated using Equation ^[7]^:

$\Delta C=\frac{C_{f}^{0}-(C_{f}^{t}-C_{d}^{t})}{\ln(\frac{C_{f}^{0}}{C_{f}^{t}-C_{d}^{t}})}$

where $C_{f}^{0}$ and $C_{f}^{t}$ are the concentrations in the feed-side compartment at time 0 and t, respectively, and $C_{d}^{t}$ is the concentration in the permeate-side compartment after a certain time (t).

The H^+^/Fe^2+^ selectivity (S) of the TBF membrane could be calculated using Equation ^[8]^:

$S=\frac{U_{H^{+}}}{U_{F\text{e}^{2+}}}$

## 6. Supplementary membrane property measurements

### 6.1 Water uptake and swelling ratio

The water uptake and area swelling ratio were determined by measuring the changes in membrane weight and surface area before and after membrane wetting. The square-shaped membrane (2 $\times$ 2 cm^2^) was initially subjected to thermal treatment at 80 °C for 24 hours. Subsequently, the dry weight (W_dry_) and dry area (A_dry_) were measured. The membrane was then immersed in water for additional 48 hours. After removing the excess surface water, the wet weight (W_wet_) and wet area (A_wet_) of the membrane were measured. Three sets of each test were performed, and the water uptake and area swelling ratio (%) were calculated using Equations ^[9]^:

$$Water\text{ }uptake\text{ }\left(\%\right)=\frac{(W_{wet}-W_{dry})}{W_{dry}}\times100\%$$

$$Area\text{ }swelling\text{ }ratio\text{ }\left(\%\right)=\frac{(A_{wet}-A_{dry})}{A_{dry}}\times100\%$$

### 6.2 Ion exchange capacity

The ion exchange capacity (IEC) values were determined via titration. Membrane samples in NaCl solution form 1M were immersed in aqueous Na_2_SO_4_ solution (0.5 M) for 24 h to release the Cl^-^ ions from membranes. The released Cl^-^ ions were titrated with an aqueous AgNO_3_ solution (C_AgNO3_ = 0.01 M) and K_2_CrO_4_ was used as the indicator. Three sets of each test were performed, and the IEC values were subsequently calculated using Equation ^[10]^:

$IEC\text{ }\left(mmol/g\right)=\frac{(V_{AgNO_{3}}\times C_{AgNO_{3}})}{m_{dry}}$

where V_AgNO3_ and m_dry_ are the volume of AgNO_3_ consumed and the weight of dry membrane samples respectively.

### 6.3 Electrochemical impedance spectroscopy (EIS)

Electrochemical Impedance Spectroscopy (EIS) was employed to measure the ohmic resistance of all membranes, using the FRA impedance potentiostatic mode with a voltage bias of 10 mV and a frequency range of 100000Hz to 0.01Hz. The membranes were saturated in solutions of 0.5 M HCl, 0.5 M NaOH or 0.5 M FeCl_2_ and subsequently installed in a self-made four-electrode device. The ohmic resistance (Rohmic) of membranes was determined by the intersection points of the EIS curves with the x-axis. The ionic conductivity of these membranes, within a temperature range from room temperature to 60°C, can be calculated according to the following equation:

$\sigma=\frac{l}{A\times R_{ohmic}}$

where 𝜎 is the ionic conductivity, 𝑙 is the membrane thickness, 𝐴 is the effective area, and R_ohmic_ is the membrane resistance. The effective membrane area is 1.766 cm^2^. The overall thicknesses of the membranes were determined by a micrometer, and the thickness of the selective layer was tested by SEM. Arrhenius plots were derived using the ionic conductivity in the temperature range of room temperature to 60 °C. The Arrhenius equation was employed to linearly fit $\ln\left( \sigma\right)-\frac{1}{T}$ plots to obtain activation energy following the equations below ^[11]^:

$\sigma=\sigma_{0}\times\exp(-\frac{E_{a}}{kT})$

$\ln\sigma=\ln\sigma_{0}-\frac{E_{a}}{kT}$

where T is the temperature, 𝜎_0_ is the pre-exponential factor, E_a_ and k are the activation energy and Boltzmann constant.

### 6.4 Zeta potential

The surface charging properties of the TBF and QA-TBF were investigated using an electrokinetic analyzer (SurPass 3, Anton Paar, Australia). A 1 mM KCl aqueous solution was used as the background electrolyte, and the measurements were conducted over a pH range of 3-10 at 25°C. The zeta potential (ζ) was calculated based on the following Helmholtz Smoluchowski Equation ^[12]^:

$\zeta=\frac{dU}{dP}\times\frac{\eta}{\varepsilon\varepsilon_{0}}\times\kappa$

where U and P denote the streaming potential and pressure, respectively; η, ε, and κ are the electrolyte viscosity, permittivity, and conductivity, respectively; ε_0_ is the vacuum permittivity.

### 6.5 Limiting current density

The Limiting current density (LCD) of QA-TBF membrane was evaluated through current-voltage (I-V) measurements with a two-compartment H-cell. The two compartments were separated with the QA-TBF membrane (surface area: 1.766 cm^2^) and filled with 30 mL of a binary mixture solution containing both NaOH and Na_2_WO_4_ concentrations of 0.5 mol·L^-1^. A salt-bridged Ag/AgCl electrode was placed in each compartment and connected to an Autolab PGSTAT302N (Metrohm AG), which supplied a direct voltage that was swept from 0 to 1.2 V at a scan rate of 0.01 V s^-1^. The corresponding electrical signals were processed with NOVA2 (Metrohm AG) to plot I-V curves. The limiting current density of the membrane was obtained from the plateau region of the I-V curve.^[13]^

### 6.6 Simulations of free volume

Initially we loaded periodic cubic cells with dimensions of 6 nm. Polymerization steps were then performed between reactive groups. To allow the structure to adapt a general Amber force field with the restrained electrostatic potential charges, we controlled a cutoff of 0.6 nm with intermediate 1ns NVT for the molecular dynamics steps. After we got an amorphous structure, the total run time was 10 ns for the equilibrium molecule dynamic simulation. Polymer fractional free volume(FFV) was estimated by the following formula:

$FFV=\frac{V_{f}}{V_{f}+V_{0}}$

where V_f_ and V_0_ are the free volume and occupied volume. The values of V_f_ and V_0_ are explored using probe with radius of 1 Å, which can also give the morphology of voids in aggregate.

# Supplementary figures and data


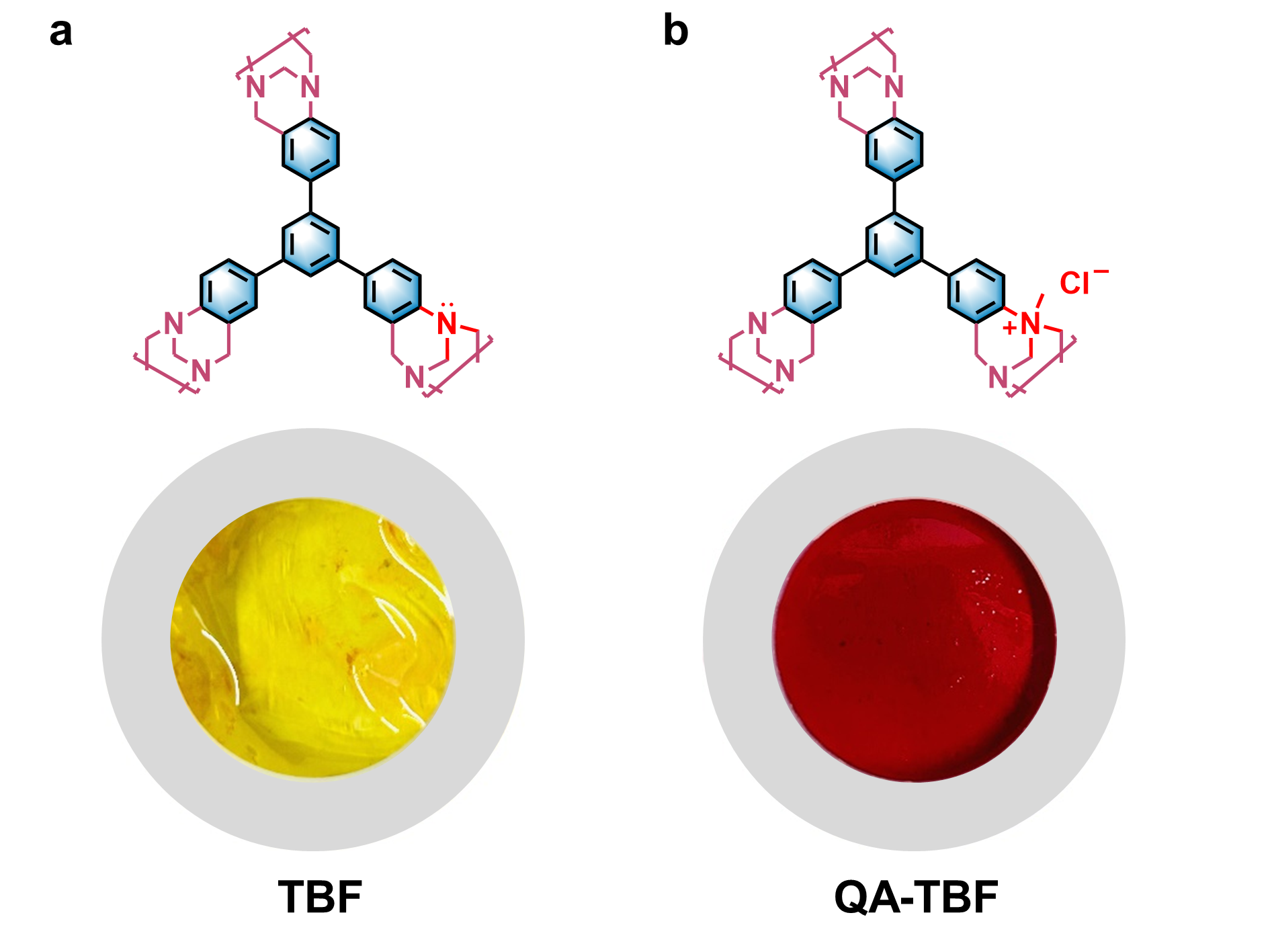


## Figure S1. Digital photo of the TBF membrane (a) and QA-TBF membrane (b).


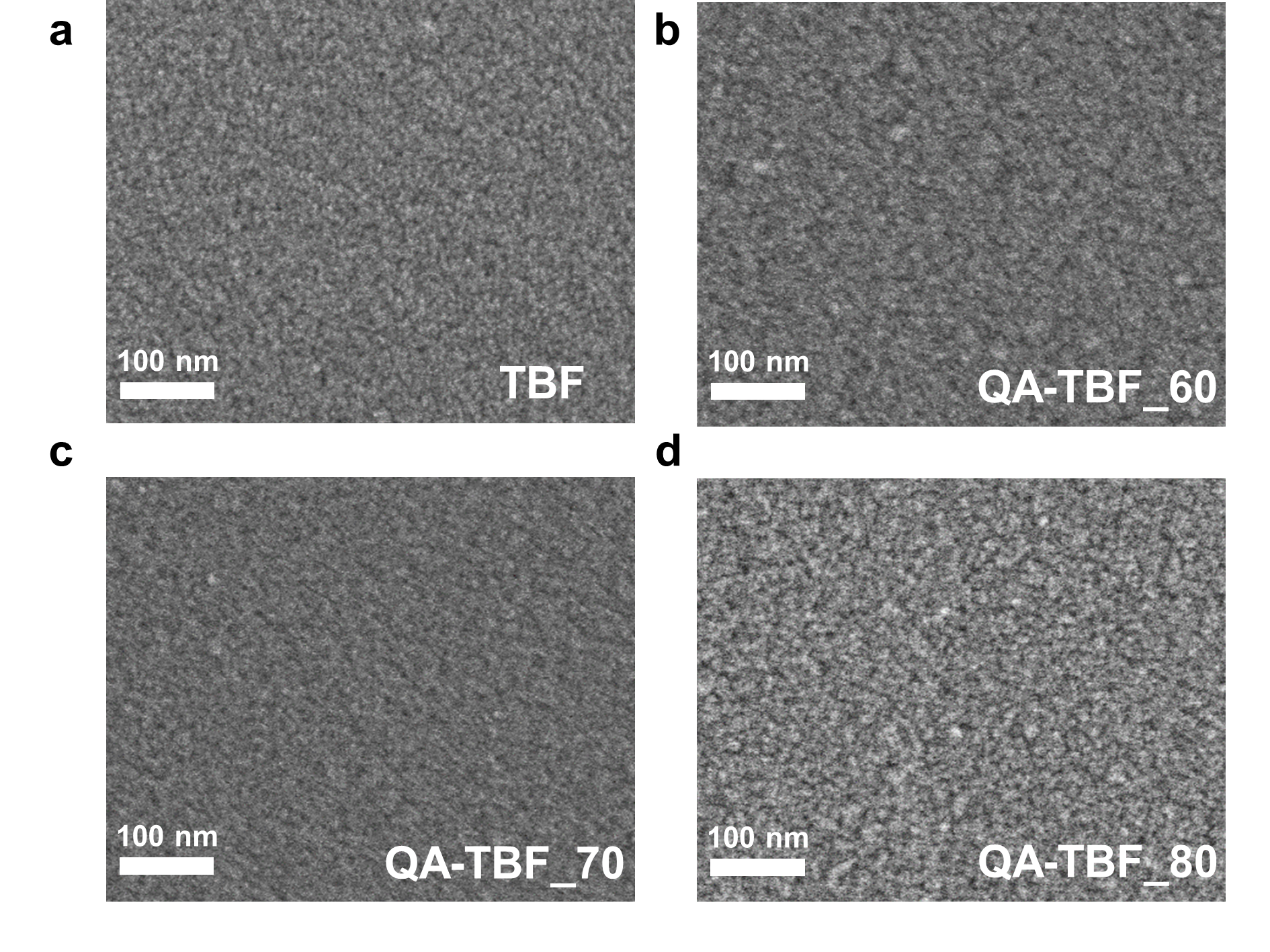


## Figure S2. SEM images illustrating the surface morphology of (a) TBF, (b) QA-TBF_60, (c) QA-TBF_70, and (d) QA-TBF_80.


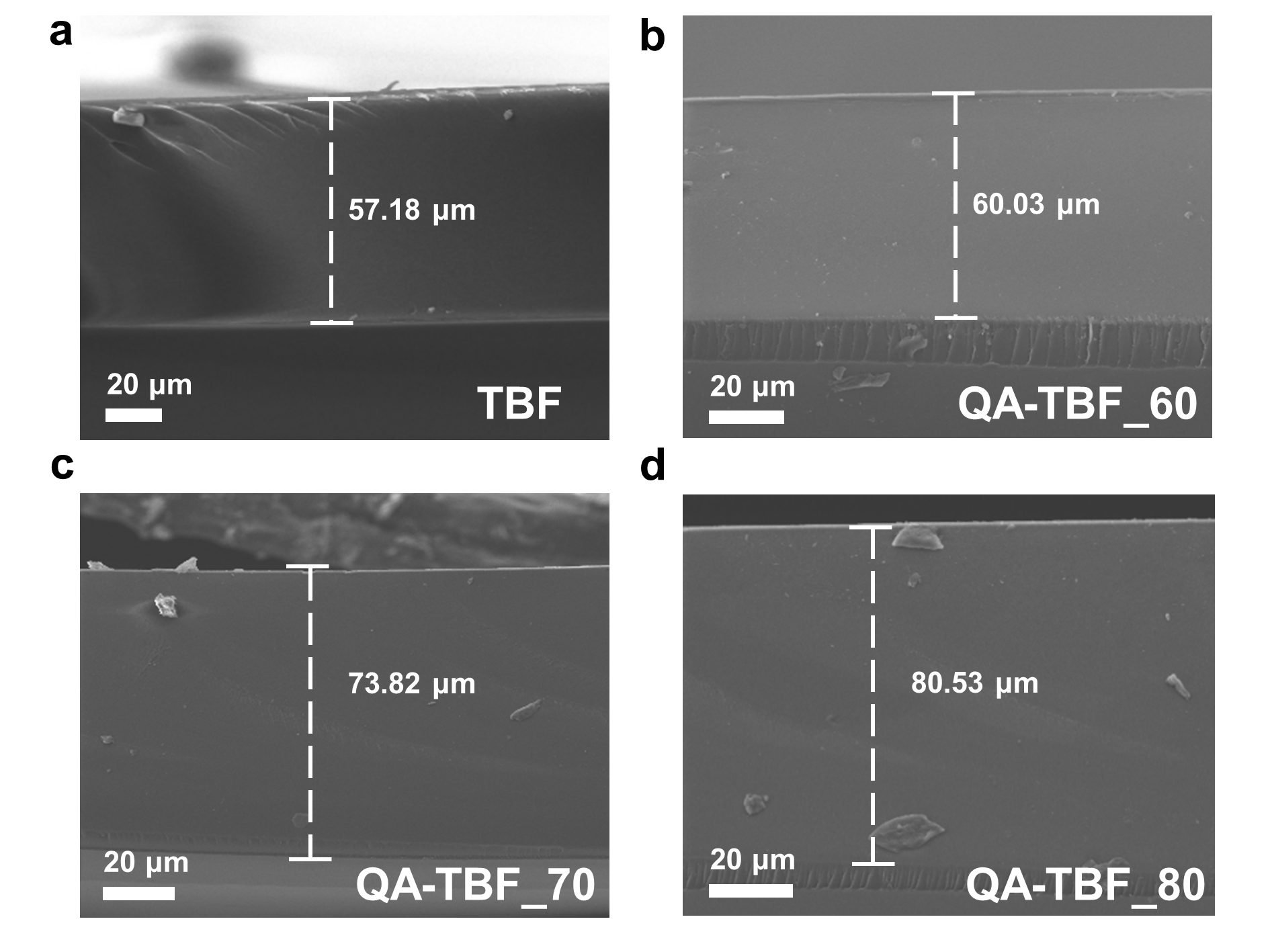


## Figure S3. Cross-sectional SEM images of (a) TBF, (b) QA-TBF_60, (c) QA-TBF_70, and (d) QA-TBF_80, revealing the internal structural features and layer compositions of each sample.


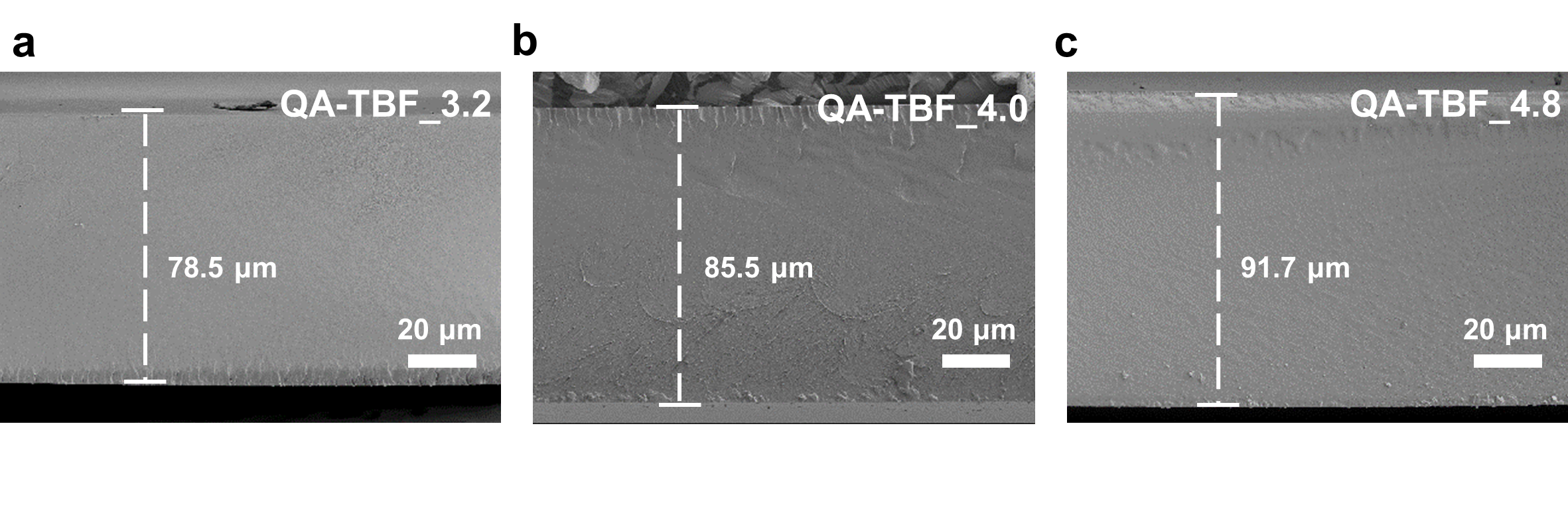


## Figure S4. Cross-sectional SEM images of (a) QA-TBF_3.2, (b) QA-TBF_4.0, and (c) QA-TBF_4.8 fabricated by varying the amount of casting solution at 3.2, 4.0, and 4.8 mL.

**Note:** For QA-TBF membranes with varying thicknesses, the monomer molar ratio (TPB:DMM = 1:6) was maintained during preparation. Using glass dishes with a fixed diameter of 60 mm, the casting solution volume was varied (3.2 mL, 4.0 mL, and 4.8 mL, corresponding to QA-TBF_3.2, QA-TBF_4.0, and QA-TBF_4.8 membranes, respectively). As the solution volume increased from 3.2 mL to 4.8 mL, the membrane thickness increased from 78.5 µm to 91.7 µm, demonstrating that membrane thickness can be effectively controlled by adjusting the casting solution volumes. Notably, no significant changes in membrane homogeneity were observed.


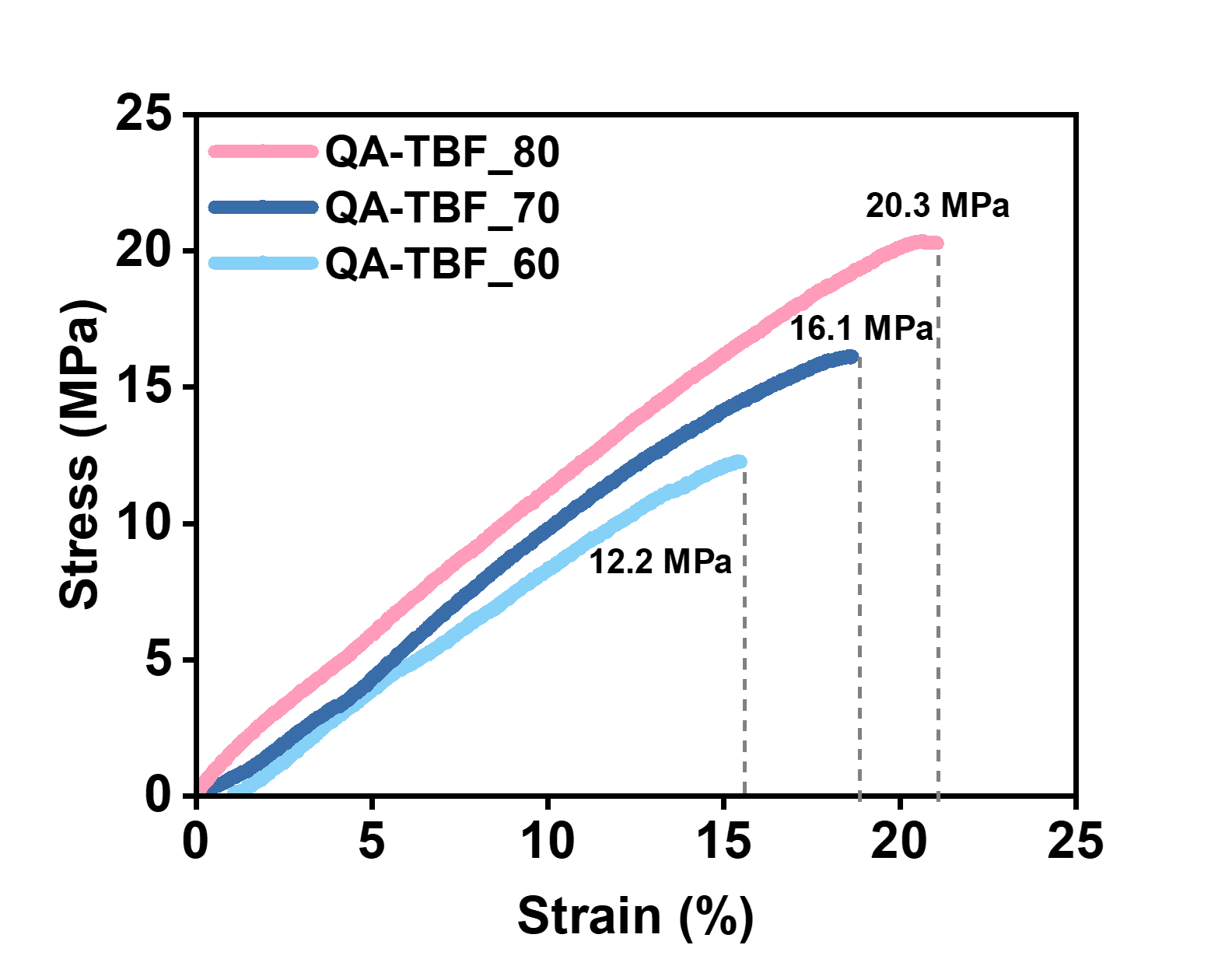


## Figure S5. Stress-strain curves of QA-TBF membranes.


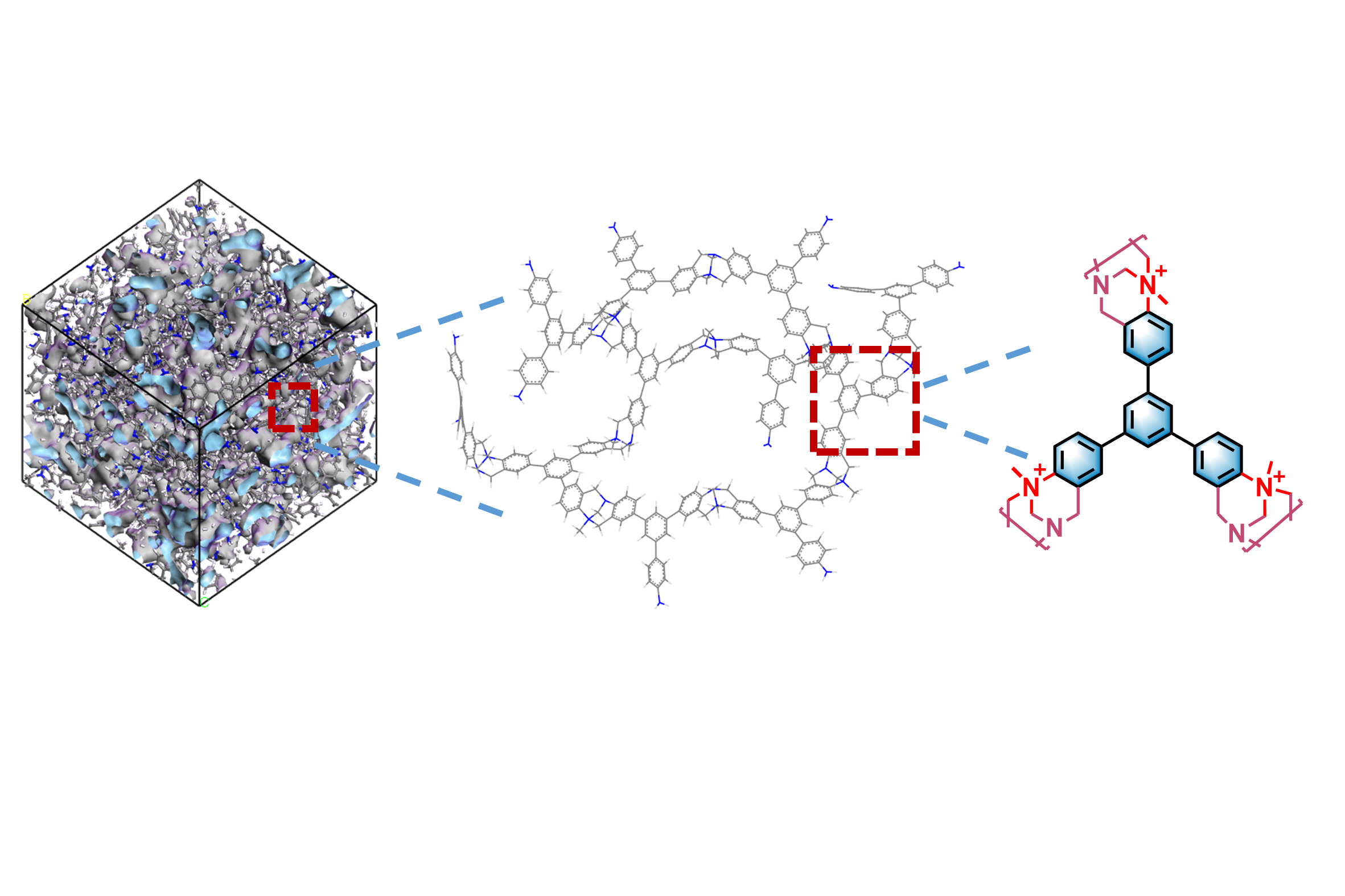


## Figure S6. The 3D representation, 2D schematic, and a depiction of a single structural unit of the QA-TBF network.


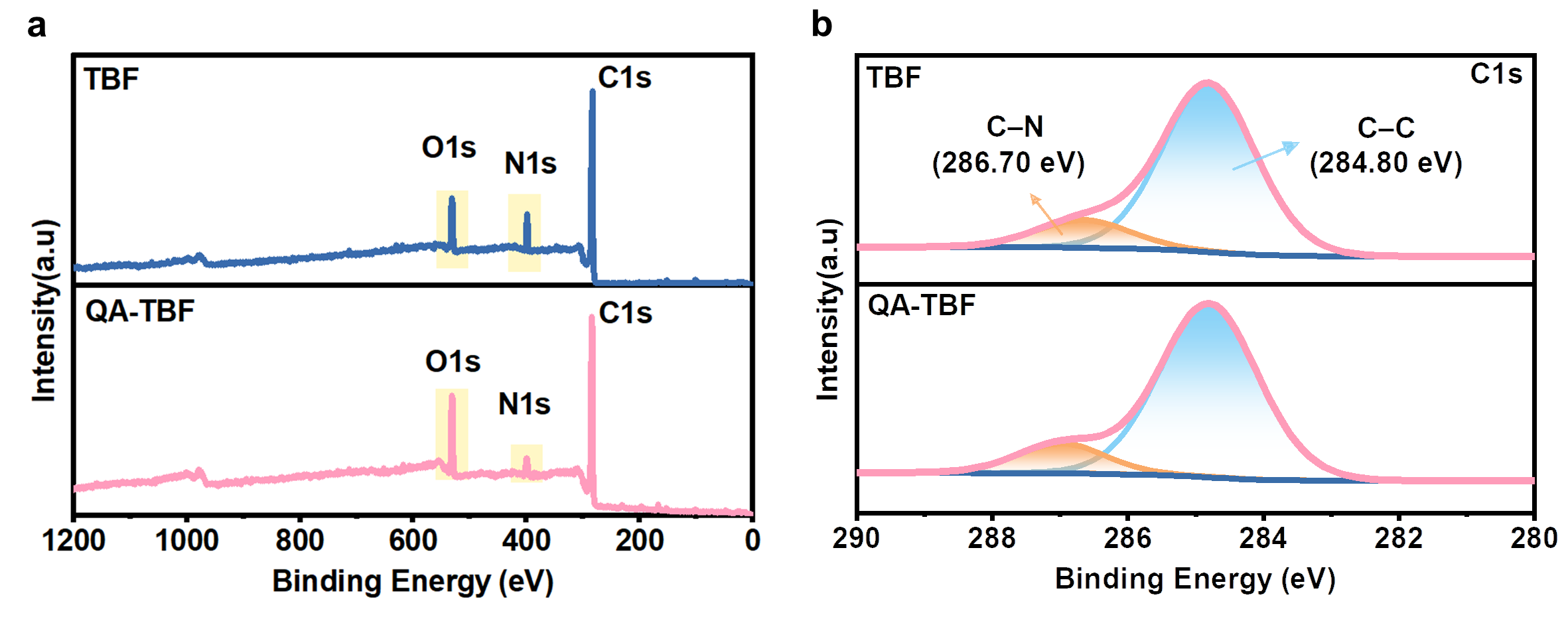


## Figure S7. (a) Full XPS spectra of TBF and QA-TBF, (b) High-resolution XPS spectra of C1s for TBF and QA-TBF.

**Note:** Upon the incorporation of quaternary amine groups into the TBF membrane and subsequent ion exchange, the changes observed in the C1s peak were minimal (from 78.66% in TBF to 75.99% in QA-TBF), indicating a stable framework. In contrast, the appearance of the N1s peak represents a significant alteration in the membrane’s ionic environment (from 9.25% in TBF to 11.62% in QA-TBF), suggesting enhanced functionalities attributable to the introduction of quaternary amine groups.


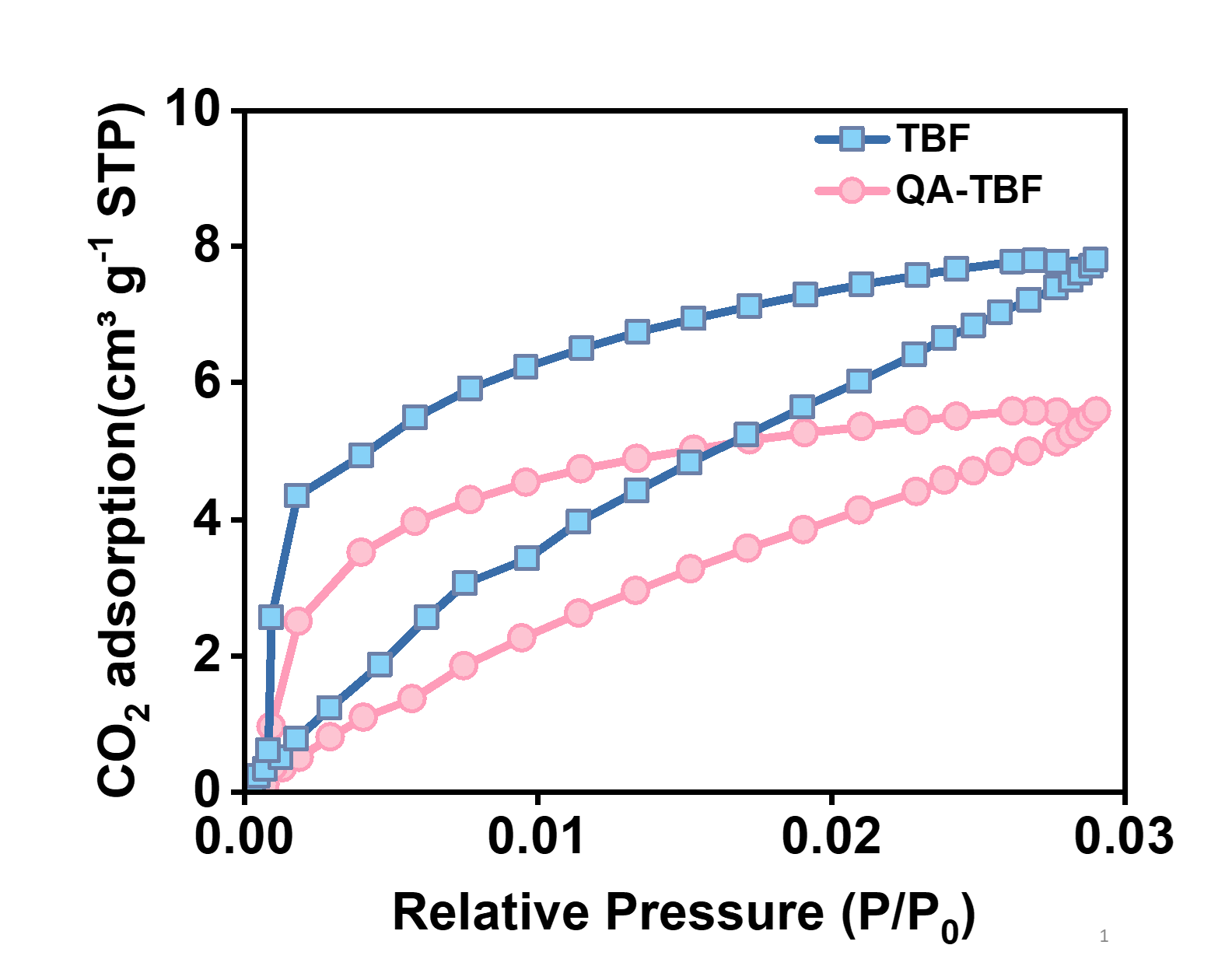


## Figure S8. CO_2_ sorption isotherms of TBF and QA-TBF at 273 K.


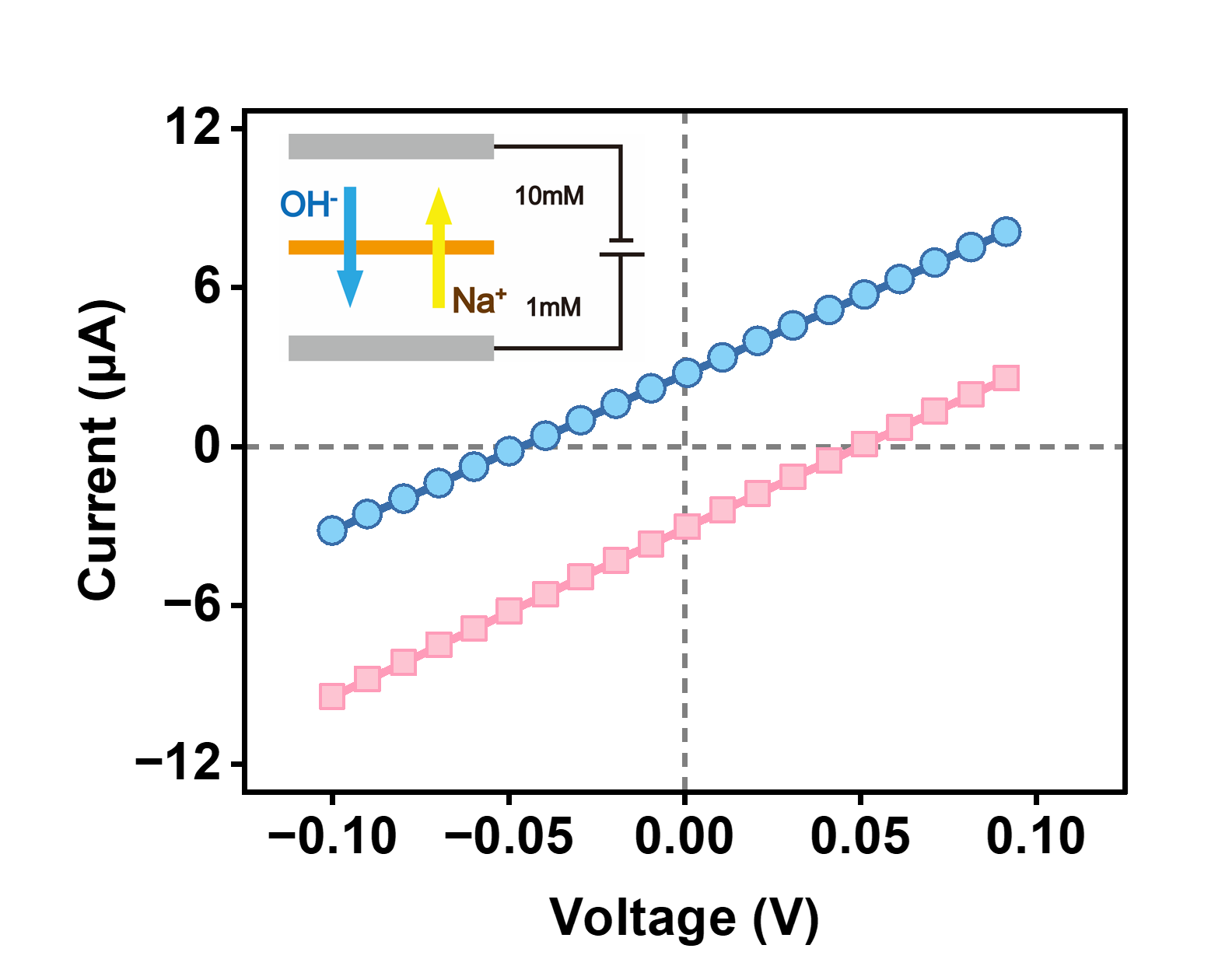


## Figure S9. Current−voltage (I−V) curves of the TBF membrane under a 10-fold concentration gradient in NaOH.

**Note:** The direction of the external voltage is in agreement with (the pink line) or opposite to (the blue line) the net anion flow from the high to the low concentration solution.


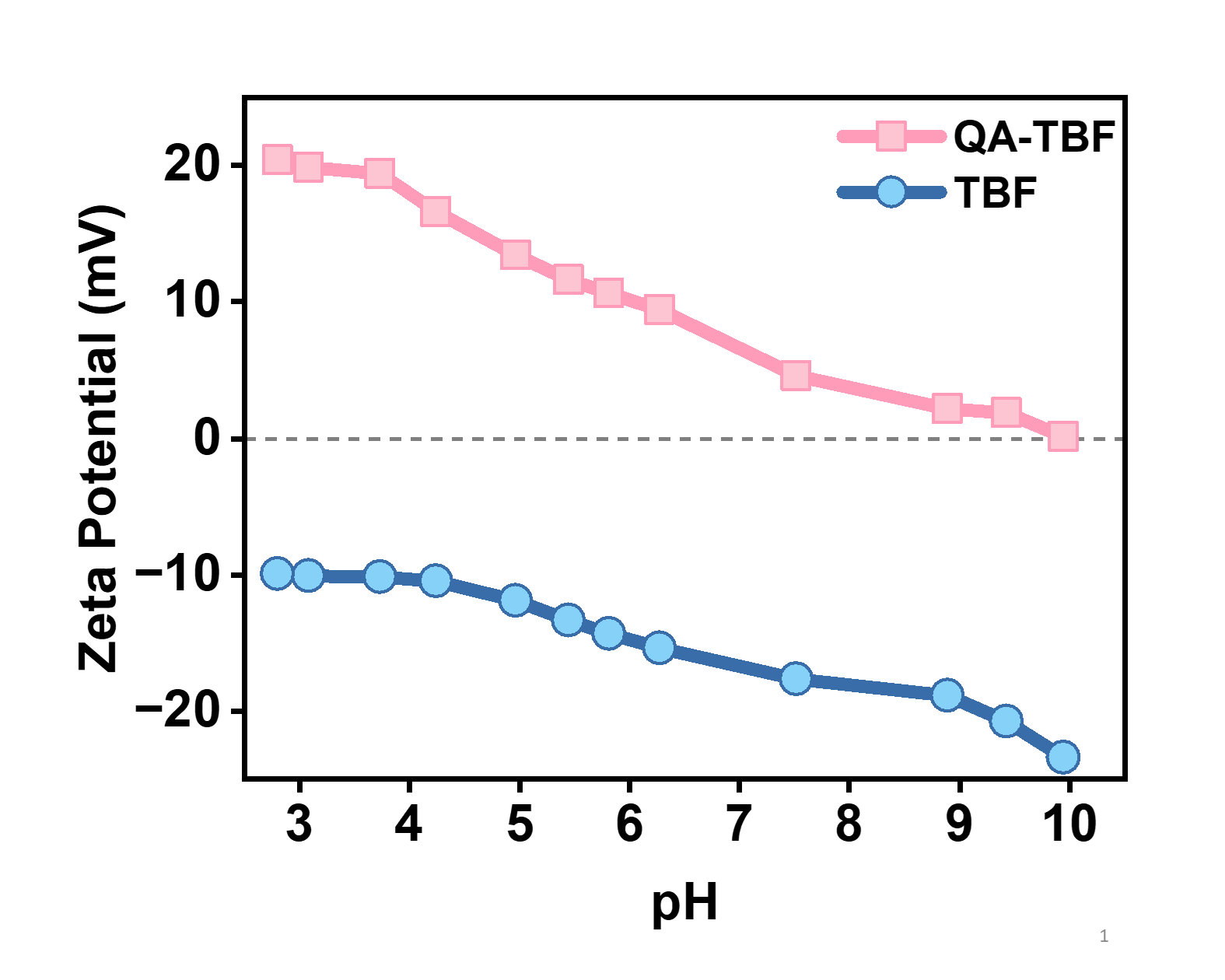


## Figure S10. Zeta Potential of TBF and QA-TBF membrane at different pH values.

**Note:** QA-TBF membranes exhibit a markedly stronger positive charge compared to TBF membranes, with zeta potentials of 19.8 mV for QA-TBF and -10.0 mV for TBF at pH 3. This pronounced positive charge is attributed to the introduction of quaternary ammonium groups, which significantly increase the density of cationic sites. Consequently, QA-TBF membranes are expected to display enhanced electrostatic interactions and improved ionic conductivity.


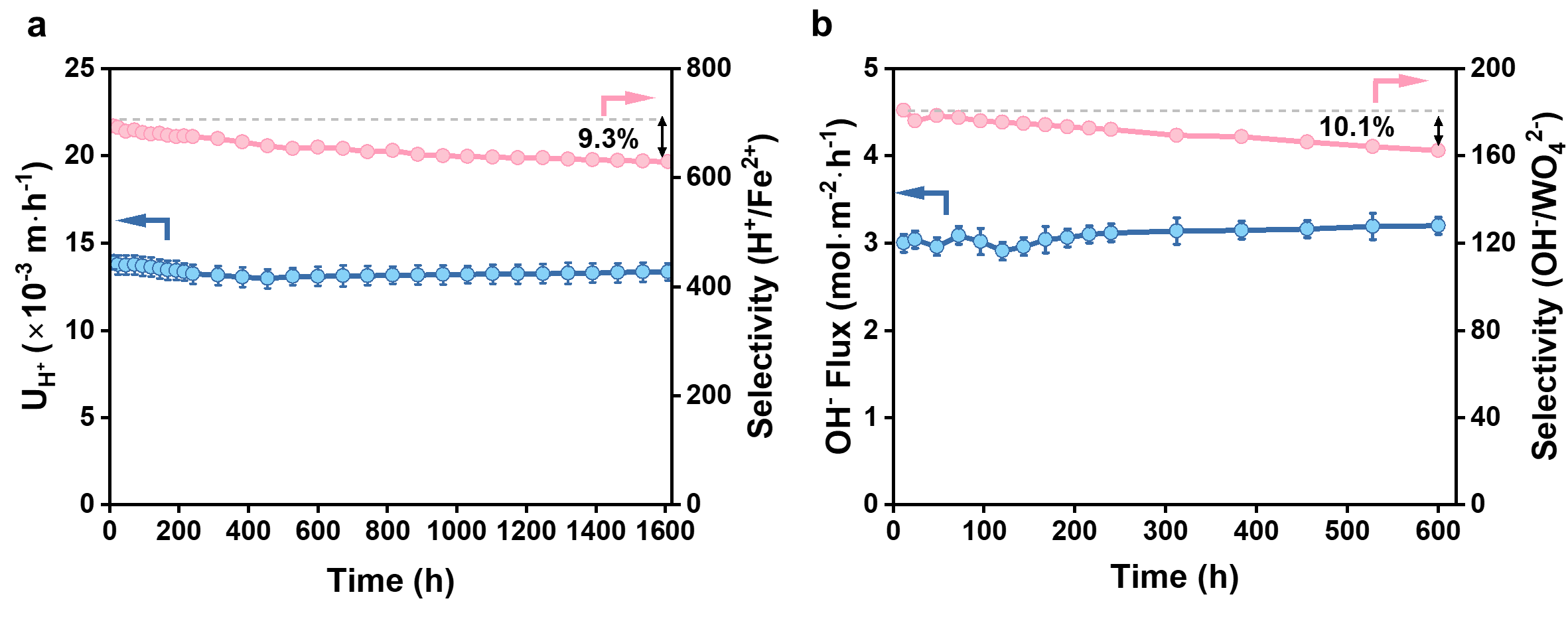


## Figure S11. (a) Diffusion dialysis acid recovery for long cycle testing. (b) Electrodialysis alkali recovery for long cycle testing.

**Note:** (a) OH⁻ flux and OH⁻/WO₄²⁻ selectivity of QA-TBF_70 membrane in mixture solution with both NaOH and Na_2_WO_4_ concentrations of 0.5 mol·L^-1^ for 600 hours at room temperature and under an applied current density of 15 mA cm⁻². (b) U_H_^+^ and H^+^/ Fe^2+^ selectivity of QA-TBF_70 membrane in mixture solution containing 0.5 mol·L^-1^ HCl and 0.2 mol·L^-1^ FeCl_2_ for 1600 hours at room temperature.


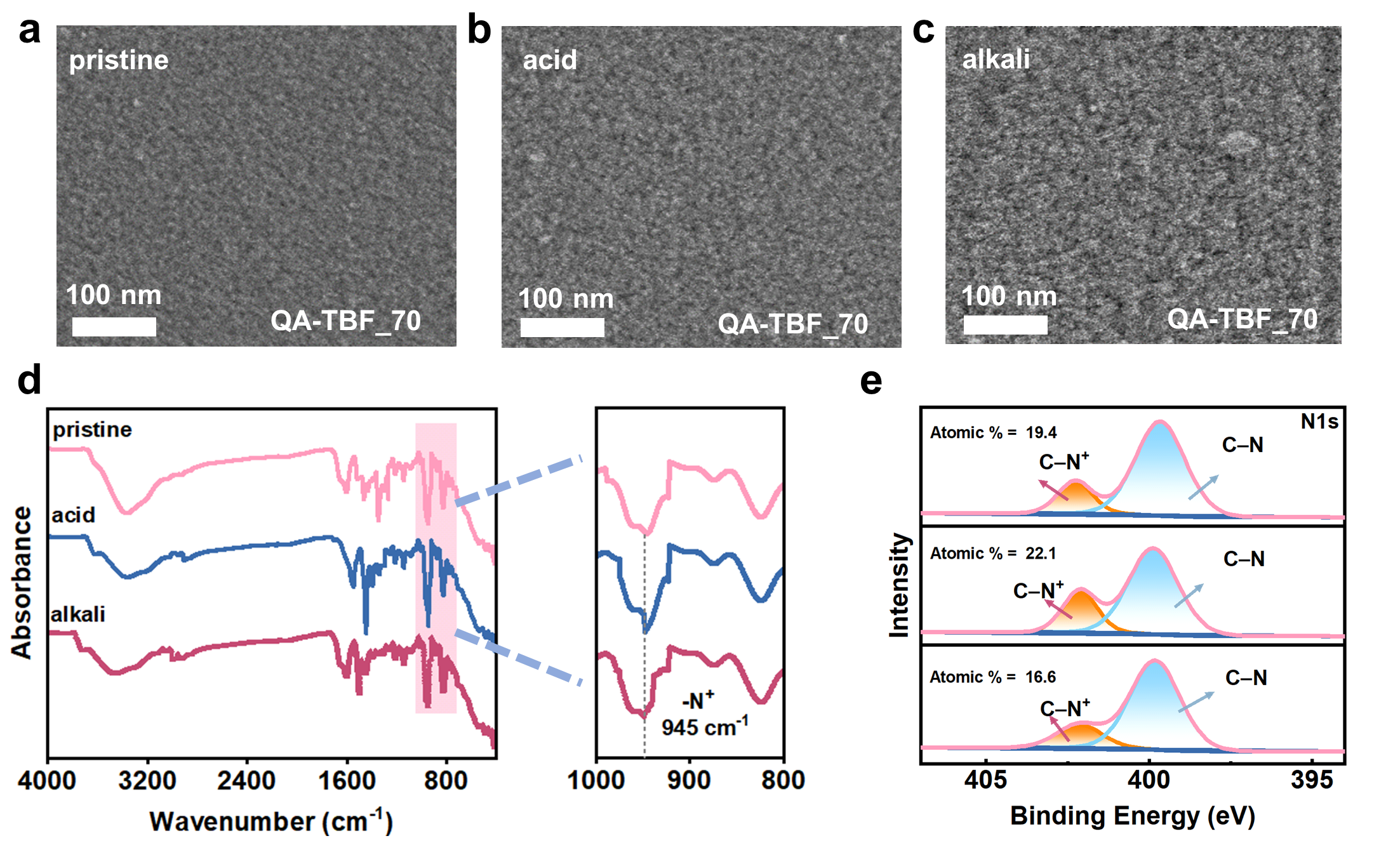


## Figure S12. SEM images illustrating the surface morphology of (a) pristine QA-TBF_70, (b) QA-TBF_70 after soaking in 0.5 M HCl solution for 600 h, (c) QA-TBF_70 after soaking in 0.5 M NaOH solution for 600 h. (d) FT-IR spectra and detailed spectrogram of QA-TBF_70 in three states. (e) N 1s core-level spectra of QA-TBF_70 in three states, with the upper representing the pristine membrane, the middle showing acid treatment, and the bottom showing alkali treatment.

**Note:** In-situ monitoring of the physicochemical properties of the QA-TBF membrane was conducted under both acidic (0.5 M) and alkaline (0.5 M) conditions. The results revealed that the QA-TBF membranes maintained remarkable stability during continuous long-term operation, exhibiting minimal changes in both physical and chemical structures compared to their initial states. These findings imply the potential of QA-TBF membranes for use in electrodialysis for alkaline recovery and diffusion dialysis for acid recovery.


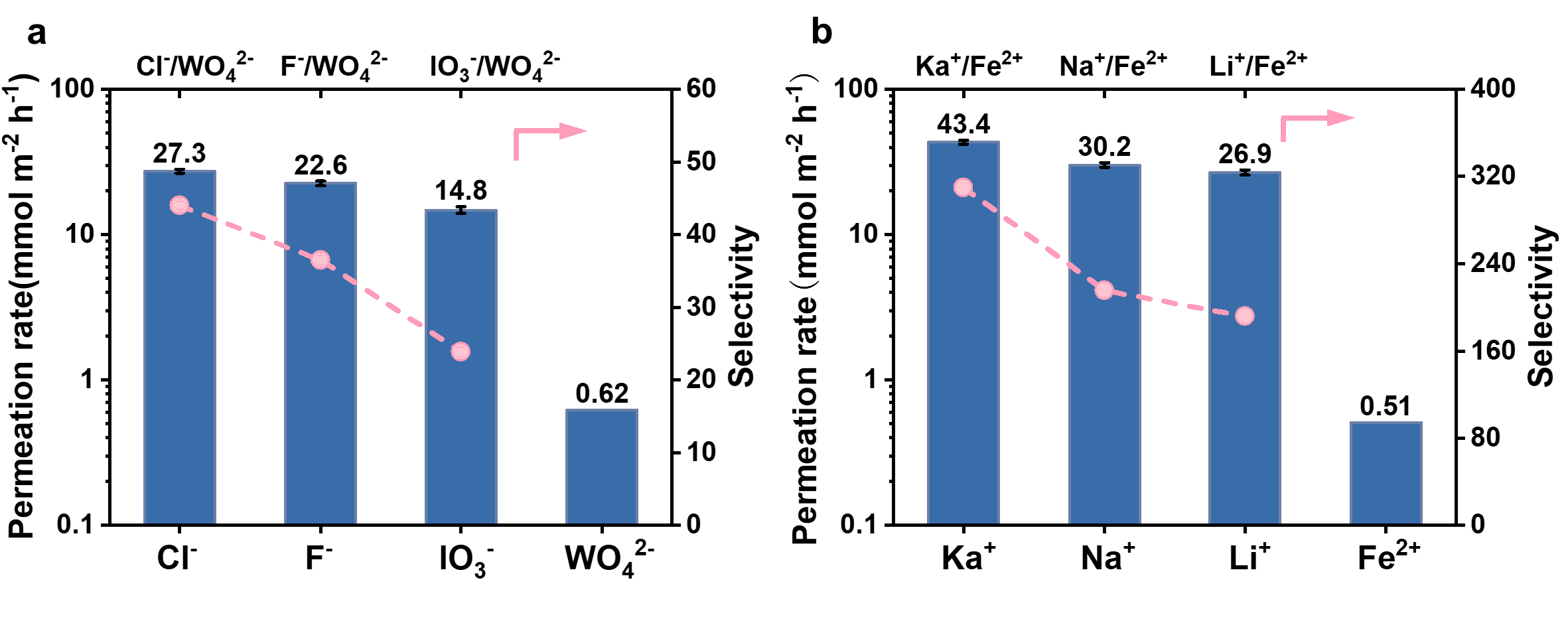


## Figure S13. Salinity concentration-driven ion-selective transporting behavior and structural characterization analysis of QA-TBF. (a) Electrodialysis diffusion behavior and monovalent (X^-^)/multivalent (WO_4_^2-^) ion selectivity in 0.2 M NaOH solution. (b) Crossover diffusion behavior and monovalent (M^+^)/multivalent (Fe^2+^) ion selectivity in 0.2 M HCl solution.

**Note:** In general, the QA-TBF membrane demonstrates excellent selective transport for target monovalent ions, with high rejection of divalent ions (Fe²⁺ and WO₄²⁻), effectively overcoming the ionic interference caused by competitive interactions among mixed ions. Under acidic conditions, the exceptional monovalent anion-selective (K^+^, Na^+^, Li^+^) transport can be attributed to the synergistic effects of size-sieving and Donnan exclusion, resulting from the presence of quaternary ammonium groups. Under alkaline conditions, excellent monovalent cation-selective (Cl^-^, F^-^, IO_3_^-^) transport is primarily governed by the size-sieving effect, wherein larger anions are restricted by angstrom-scale channels.


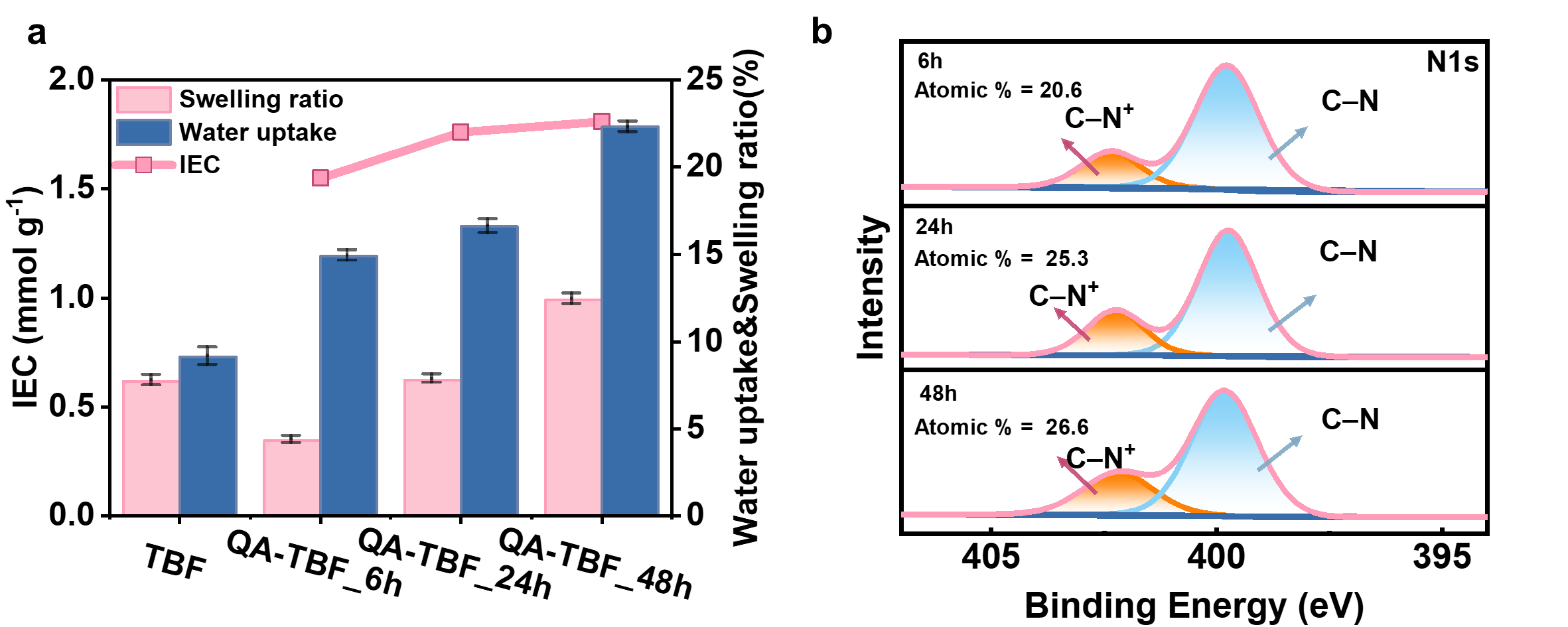


## Figure S14. (a) Water uptake and swelling ratio of TBF and QA-TBF membranes as a function of ion exchange capacity (IEC). (b) N 1s core-level spectra of TBF with different degrees of quaternization.

**Note:** The quaternization degree can be increased by prolonging the quaternization treatment duration, as demonstrated in Figure S14(b). A higher degree of quaternization increases the density of positively charged sites with high polarity, which enhances anion conductivity but may also cause excessive swelling and reduced mechanical stability, as evidenced by the rapid increase in water uptake (WU) and swelling ratio (SR) shown in Figure S14(a). Additionally, higher quaternization degrees may also reduce the free volume due to tighter polymer packing induced by the increased density of ionic groups as evidenced in Figure 1h, which is not conducive to efficient acid/alkali recovery. Considering this trade-off, QA-TBF_24h was selected as the optimal choice to achieve a balance between ionic conductivity and structural stability.


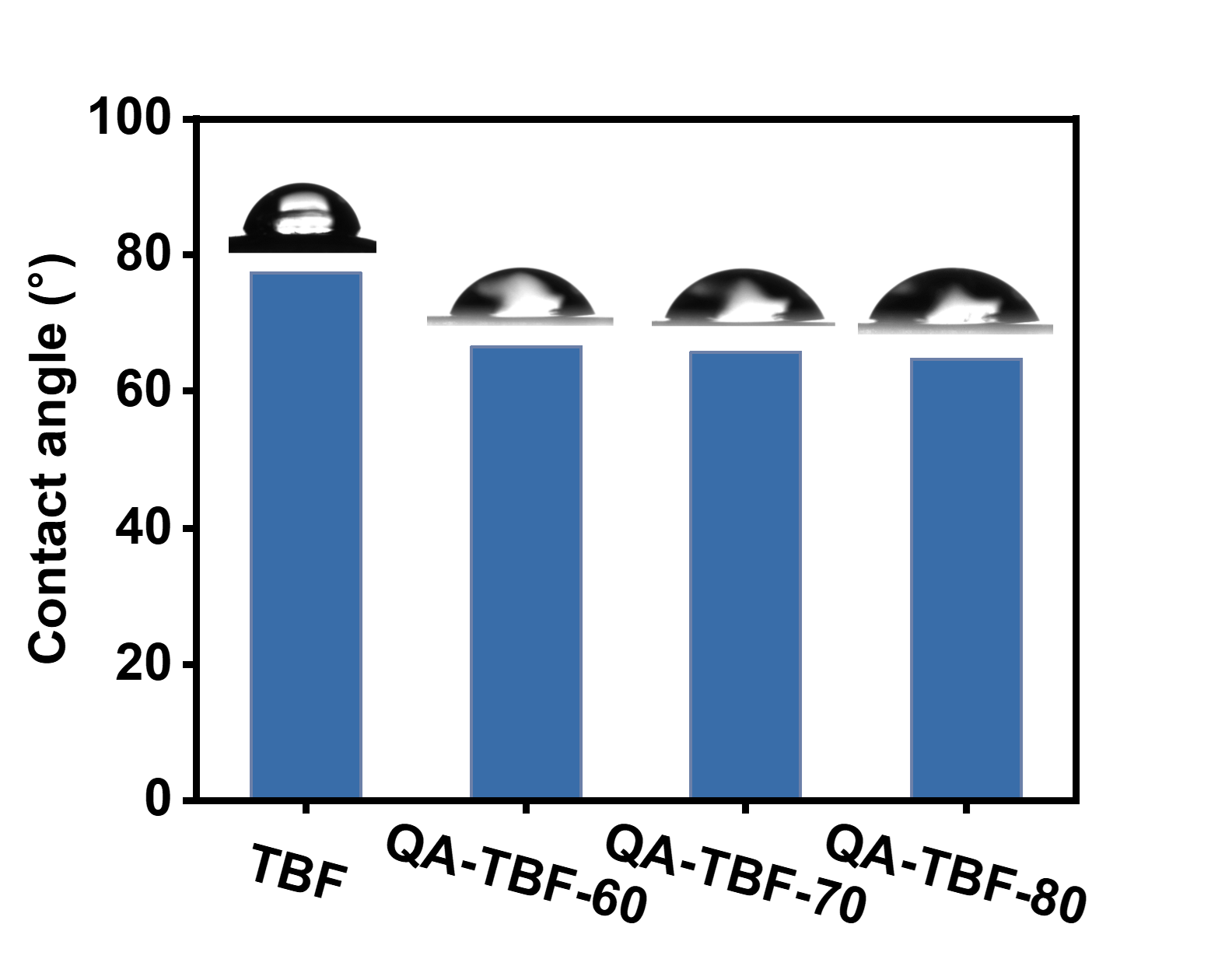


## Figure S15. Contact angle of TBF and QA-TBF membranes.

**Note:** The water contact angle of QA-TBF membranes decreased from 77.5° for the original TBF membranes to 66.5°, 65.75°, and 64.75°, respectively.


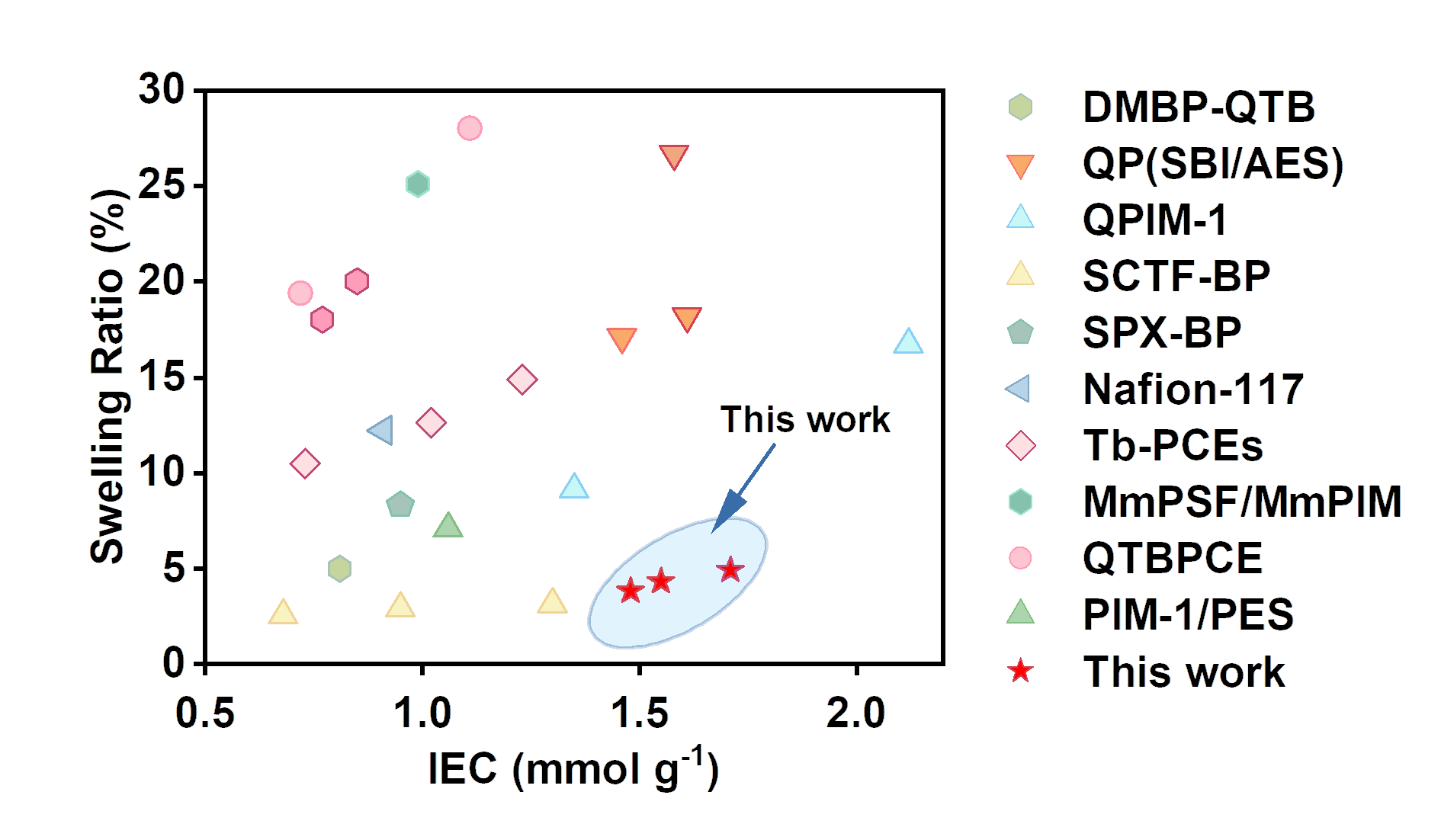


## Figure S16. Swelling ratio as a function of ion exchange capacity (IEC) for QA-TBF and other reported membranes, with detailed values presented in Table S1 for reference.


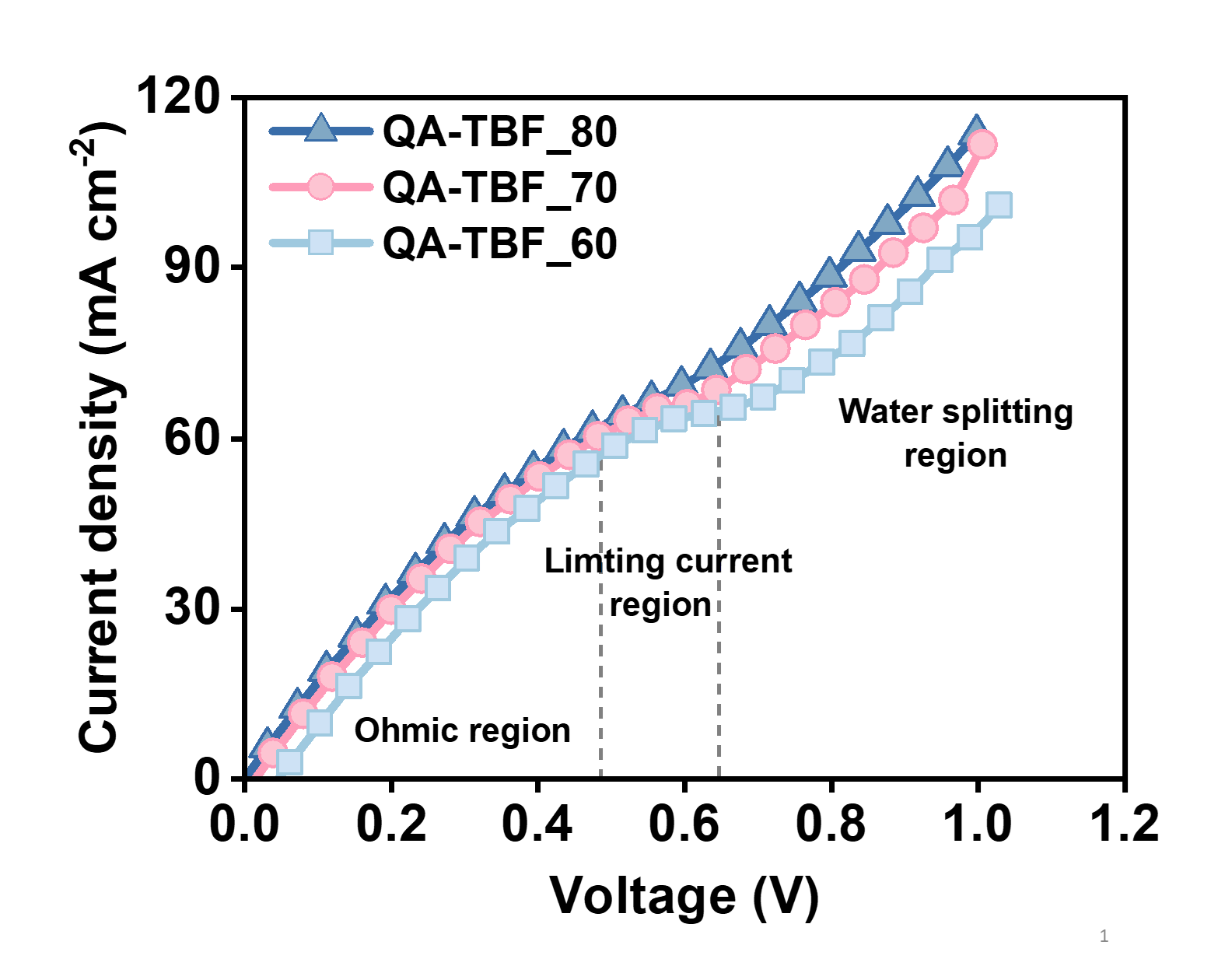


## Figure S17. Current-voltage (I-V) curves of QA-TBF membranes with varying thicknesses.

**Note:** Analysis of the limiting current region of the I-V curve reveals that the QA-TBF membrane demonstrates a limiting current density in the range of 65 to 70 mA/cm². This observation indicates that the membrane maintains stable ion transport properties even under high current conditions, underscoring its potential for effective performance in electrochemical applications.


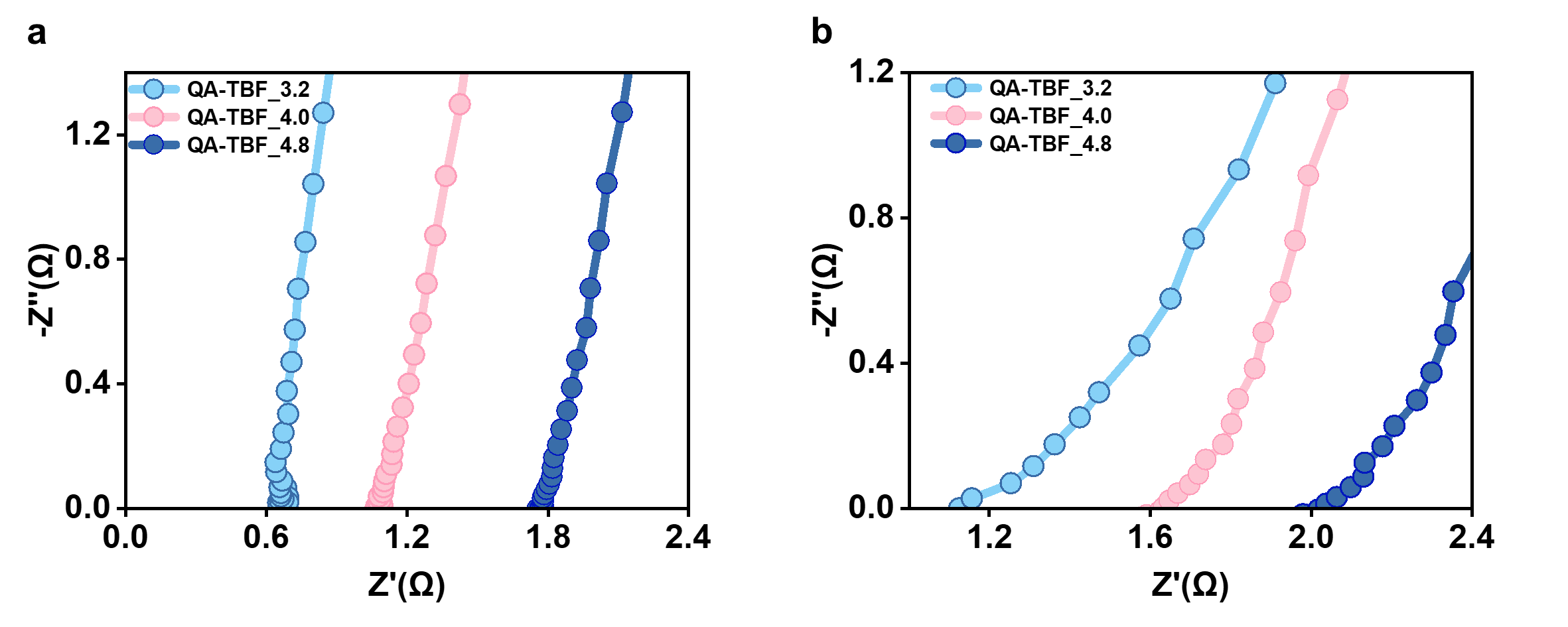


## Figure S18. EIS spectra of QA-TBF membranes fabricated using different amounts of casting solutions in (a) 0.5 M HCl and (b) 0.5 M NaOH.

**Note:** The OH⁻ and H⁺ ion transport resistance of the QA-TBF membranes was measured using electrochemical impedance spectroscopy (EIS). Compared to QA-TBF_3.2, thicker membranes exhibited progressively higher ohmic resistance. Specifically, in 0.5 M NaOH solutions, the resistance was calculated as 1.98, 2.88, and 3.53 Ω·cm², while in 0.5 M HCl solutions, it was 1.15, 1.89, and 3.11 Ω·cm² for QA-TBF_3.2, QA-TBF_4.0, and QA-TBF_4.8, respectively.


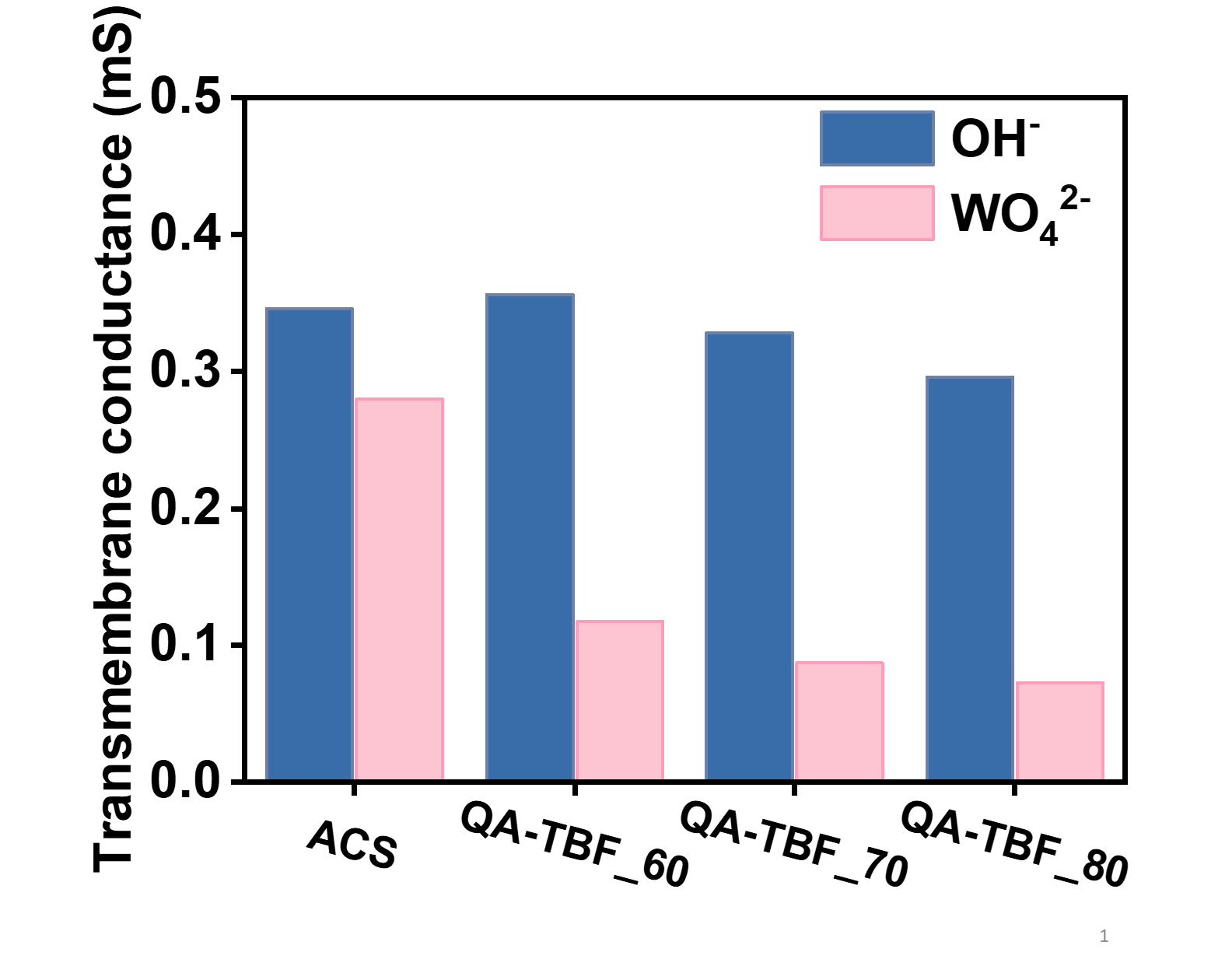


## Figure S19. Transmembrane anion conductance (OH⁻ and WO₄²⁻) plotted against the ACS (ASTOM) and QA-TBF membranes with varying thicknesses.


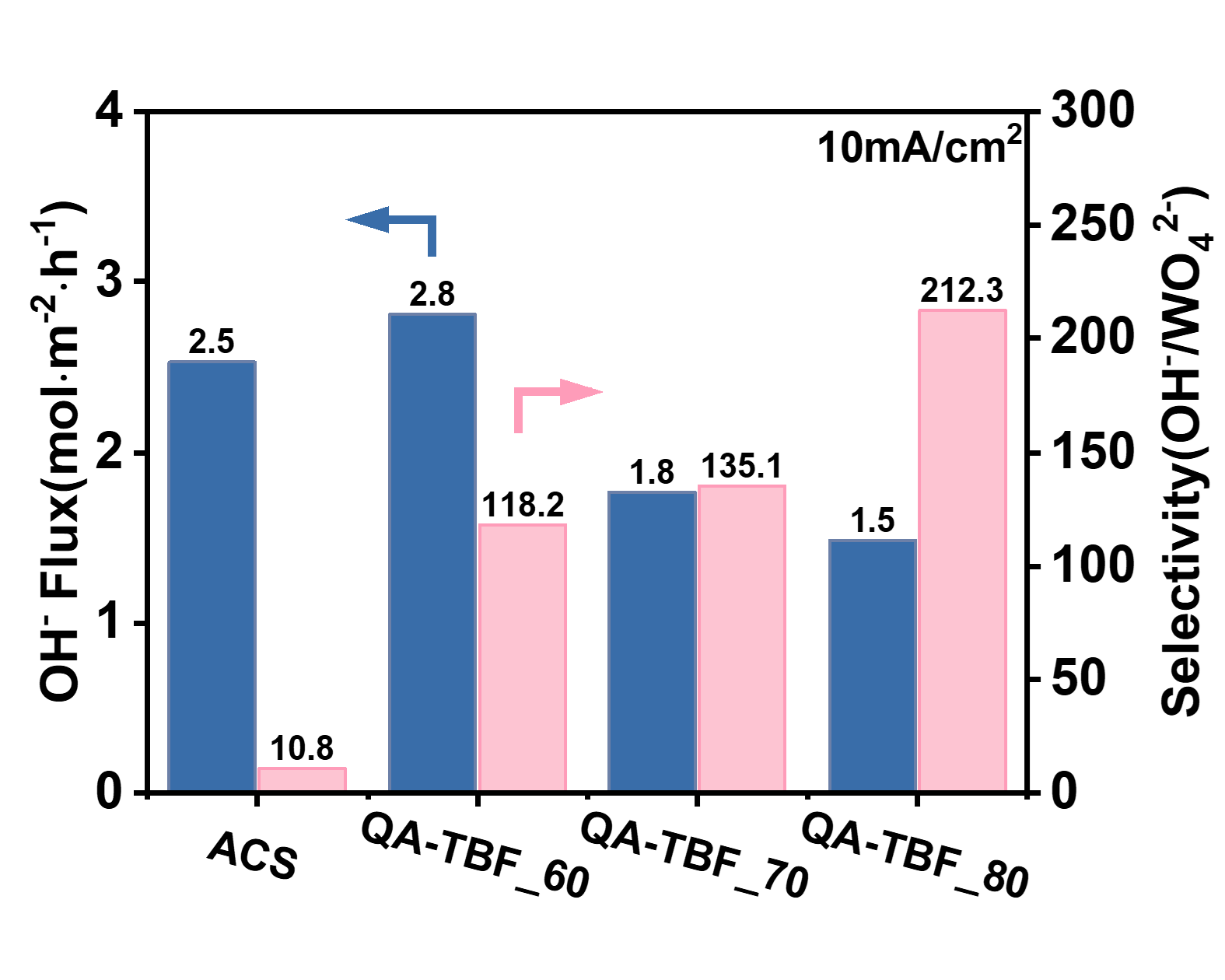


## Figure S20. OH⁻ flux and OH⁻/WO₄²⁻ selectivity of ACS (ASTOM) and QA-TBF membranes with varying thicknesses, measured at a current density of 10 mA cm⁻².


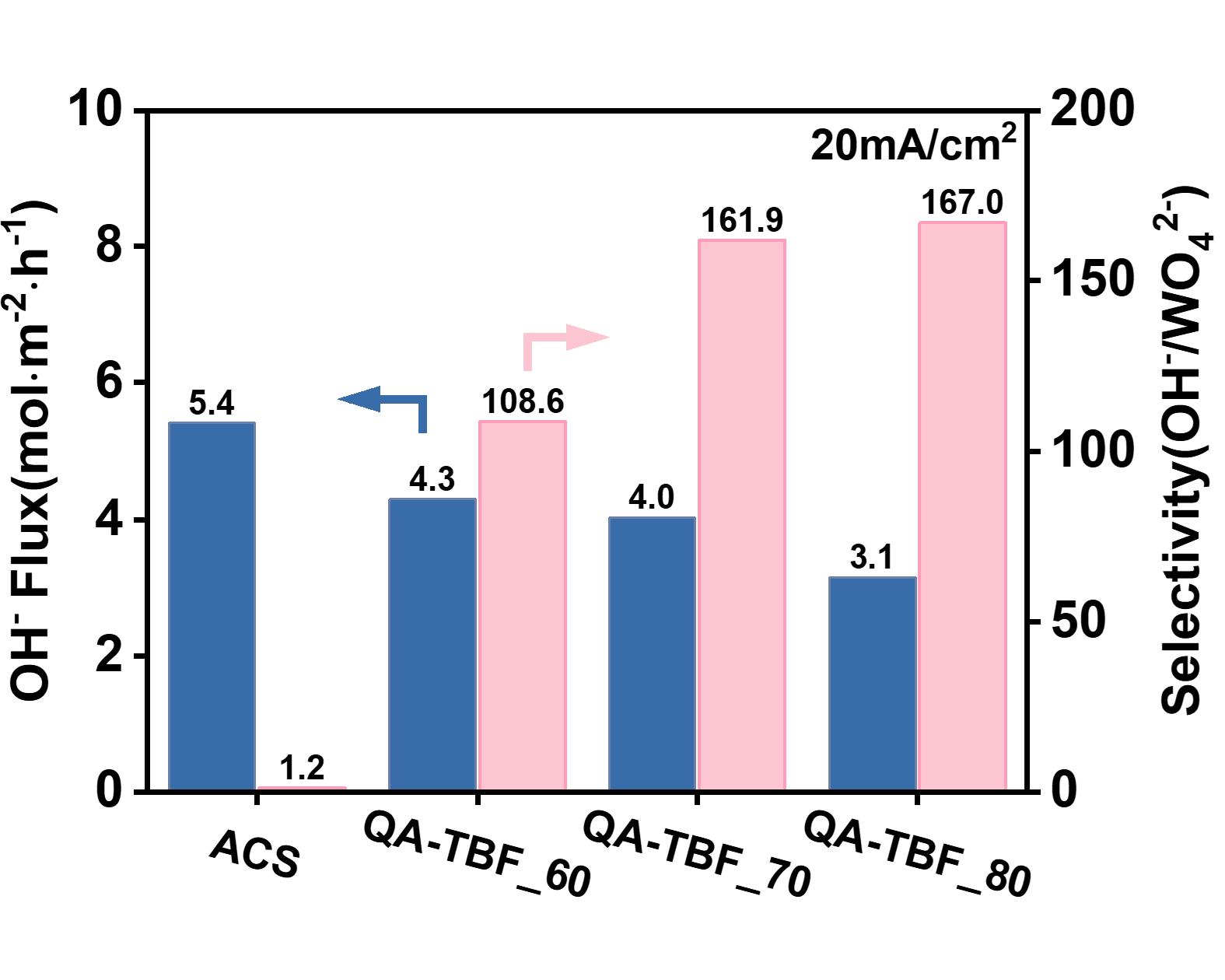


## Figure S21. OH⁻ flux and OH⁻/WO₄²⁻ selectivity of ACS (ASTOM) and QA-TBF membranes with varying thicknesses, measured at a current density of 20 mA cm⁻².


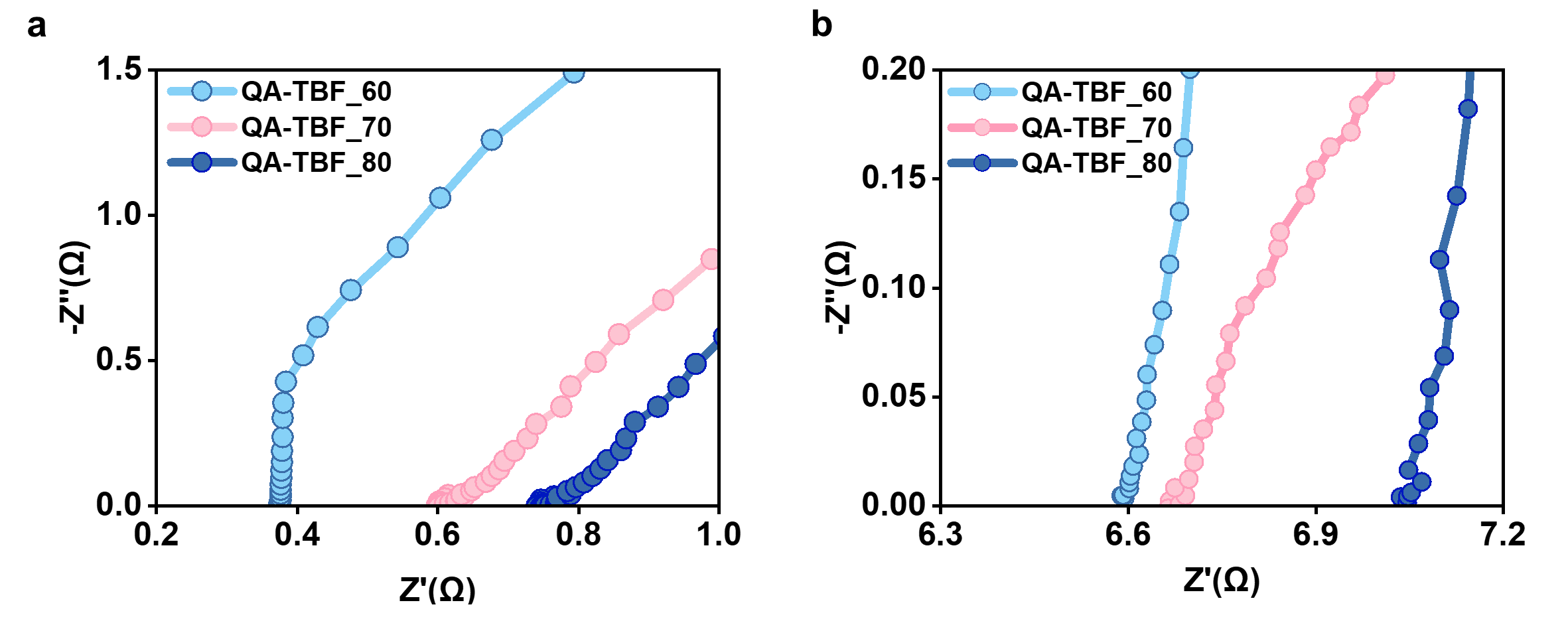


## Figure S22. EIS spectra of QA-TBF membranes with varying thicknesses in (a) 0.5 M HCl and (b) 0.5 M FeCl₂.


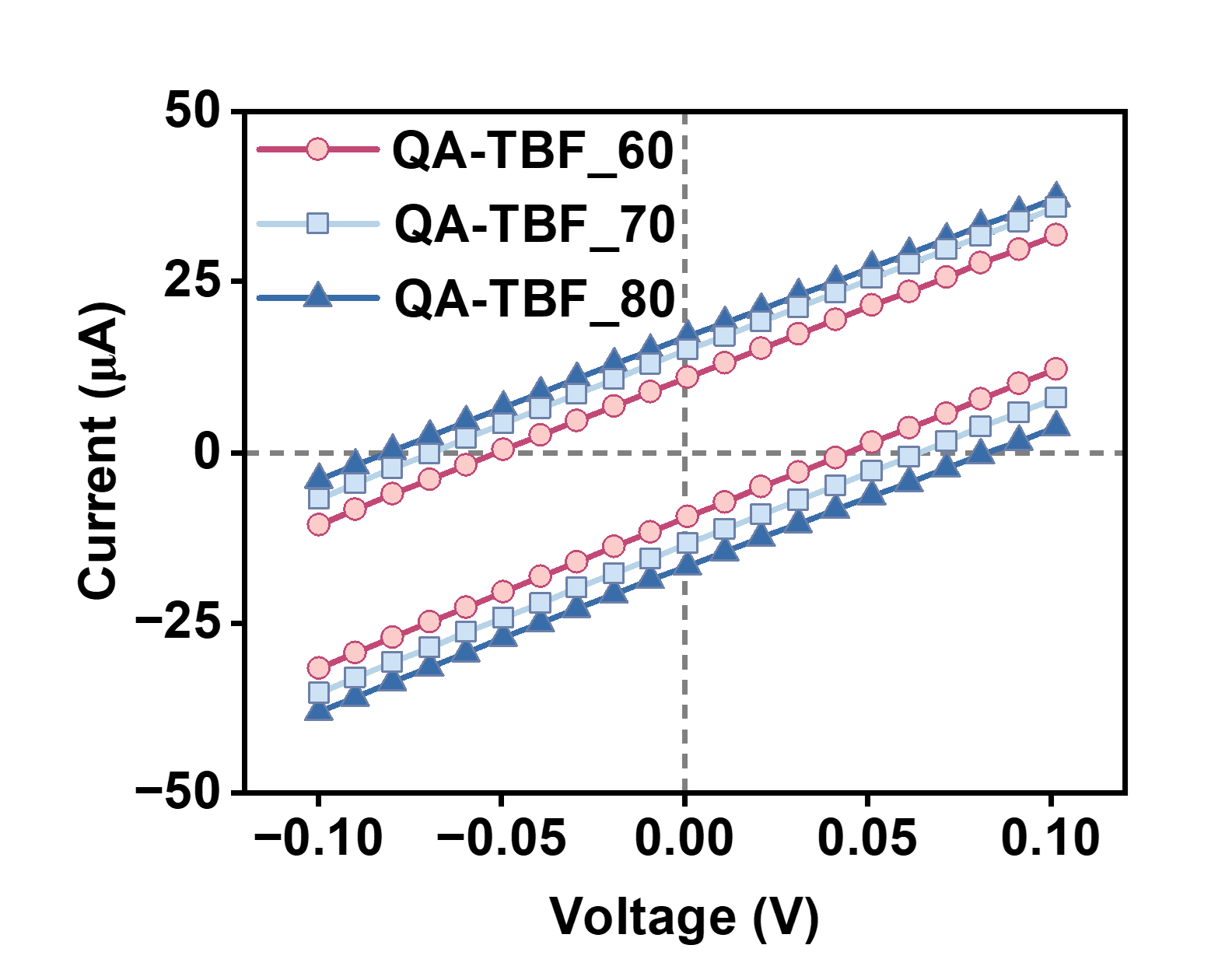


## Figure S23. Current−voltage (I−V) curves of QA-TBF membranes subjected to a 10-fold concentration gradient of KCl.

# Supplementary Tables

## Table S1. Swelling ratio (SR) as a function of IEC value for membranes derived from polymer frameworks, PIMs, and the commercial Nafion membrane.

| **Membranes** | **Structure** | **IEC (mmol g^-1^)** | **SR(%)** | **Ref** |
| --- | --- | --- | --- | --- |
| QA-TBF | Polymer Framework | 1.46  1.55  1.72 | 11.6  13.3  16.2 | This  work |
| QP(SBI/AES)-0 | PIM | 1.46 | 17.10 | [14] |
| QP(SBI/AES)-1 |  | 1.61 | 18.20 |  |
| QP(SBI/AES)-0.75 |  | 1.58 | 26.70 |  |
| SPK/GO-1 | Composite membrane | 1.45 | 6.3 | [15] |
| SPK/GO-3 |  | 1.52 | 7.8 |  |
| SPK/GO-5 |  | 1.61 | 9.9 |  |
| SCTF-BP | Polymer Framework | 0.68  0.95  1.30 | 2.56  2.89  3.13 | [16] |
| QPIM-1(1.4) | PIM | 1.35 | 9.10 | [17] |
| QPIM-1(2.1) |  | 2.12 | 16.70 |  |
| Tb-PCEs-1 | PIM | 1.23 | 14.90 | [18] |
| Tb-PCEs-0.75 |  | 1.02 | 12.60 |  |
| Tb-PCEs-0.5 |  | 0.73 | 10.50 |  |
| QTPBCE | PIM | 1.11 | 28.00 | [19] |
|  |  | 0.72 | 19.40 |  |
| Nafion-117 | PFSA | 0.91 | 12.20 | [20] |
| SPX-BP-0.95 | PIM | 0.95 | 8.30 | [20] |
| MmPSF/MmPIM-10% | PIM | 0.99 | 25.10 | [21] |
| MmPSF/MmPIM-20% |  | 0.85 | 20.00 |  |
| MmPSF/MmPIM-30% |  | 0.77 | 18.00 |  |
| PIM-1/PES | PIM | 1.06 | 7.10 | [22] |

## Table S2. Summary of membranes used in the electrodialysis-based alkali recovery.

| **Membranes** | **OH^-^ Flux**  **(mol m^-2^ h^-1^)** | **Selectivity (OH^-^/WO_4_^2-^)** | **Current Density (mA cm^-2^)** | **Ref** |
| --- | --- | --- | --- | --- |
| QA-TBF-60 | 3.39 | 114.5 | 15 | This  work |
| QA-TBF-70 | 3.04 | 181 |
| QA-TBF-80 | 2.12 | 200 |
| PVA-QGO-5 | 1.71 | 136.6 | 50 | [23] |
| PVA-QPEI-QGO-5 | 1.77 | 47.2 | 50 |
| QPC-Pip-60 | 3.52 | 70 | 10 | [24] |
| QPPO-C6-120 | 9.79 | 76 | 30 | [25] |
| QPAB-2 | 3.58 | 150 | 10 | [4] |
| AMV | 1.63 | 11.1 | 50 | [23] |
| ACS | 3.12 | 1.9 | 10 | [24] |
| ASV | 10.51 | 12 | 30 | [25] |

## Table S3. Summary of membranes used in the diffusion dialysis-based acid recovery.

| **Membranes** | $\mathbf{U}_{\mathbf{H}^{\mathbf{+}}}$**(×10^-3^ m h^-1^)** | **Selectivity (H^+^/Fe^2+^)** | | **Feed**  **Solution** | **Ref** |
| --- | --- | --- | --- | --- | --- |
| QA-TBF-70 | 13.91 | | 694.4 | 0.36 M HCl/0.2 M FeCl_2_ | This  work |
| QA-TBF-80 | 11.70 | | 954.3 |
| Nafion 117 | 0.54 | | 370.1 |
| QPAES-DB50 | 18.92 | | 127.2 | 1.0 M HCl/0.1 M FeCl_2_ | [26] |
| DCM-1.43(30°C) | 15 | | 75 | 1.4 M HCl/0.15 M FeCl_2_ | [27] |
| DPPO-4h | 66 | | 96 | 1.0 M HCl/0.2 M FeCl_2_ | [28] |
| AEM2-Bu-2 | 47 | | 130 | 1.0 M HCl/0.18 M FeCl_2_ | [8] |
| semi-IPNs | 16.3 | | 38.6 | 1 M HCl/0.2 M FeCl_2_ | [29] |
| Poly(DMAEM- c o -γ -MPS) | 16 | | 87.7 | 1.28 M HCl/0.2 M FeCl_2_ | [30] |
| MDMH-QPPO-1 | 5.19 | | 390 | 1 M HCl/0.25 M FeCl_2_ | [31] |
| PAN-co-PVlm | 77 | | 55 | 1 M HCl/0.18 M FeCl_2_ | [8] |
| BPPO-B | 37 | | 351 | 1 M HCl/0.21 M FeCl_2_ | [32] |
| BPPO-Im/ GO | 29.3 | | 115 | 1 M HCl/0.21 M FeCl_2_ | [33] |
| QPPO-DM | 3.9 | | 1391 | 1 M HCl/0.27 M FeCl_2_ | [6] |
| U-C(60)/QPPO | 8.9 | | 565 | 1 M HCl/0.2 M FeCl_2_ | [34] |
| MOF-808@QPPO | 17.95 | | 435 | 1 M HCl/0.25 M FeCl_2_ | [35] |
| UiO-66@QPPO | 15.9 | | 680 | 1 M HCl/0.25 M FeCl_2_ | [35] |
| TbTGCl/CPAN | 6.1 | | 1266 | 1 M HCl/0.26 M FeCl_2_ | [36]  [36] |
| TpTGCl/CPAN | 9.5 | | 492.1 | 1 M HCl/0.26 M FeCl_2_ |

# Reference

[1] C. Guizard, A. Ayral, M. Barboiu, A. Julbe, in *Handbook of Sol-Gel Science and Technology: Processing, Characterization and Applications*, Springer International Publishing, **2018**, pp. 1971-2017.

[2] J. Lu, H. Zhang, J. Hou, X. Li, X. Hu, Y. Hu, C. D. Easton, Q. Li, C. Sun, A. W. Thornton, M. R. Hill, X. Zhang, G. Jiang, J. Z. Liu, A. J. Hill, B. D. Freeman, L. Jiang, H. Wang, *Nat Mater* **2020**, *19*, 767-774.

[3] J. Kamcev, D. R. Paul, G. S. Manning, B. D. Freeman, *Macromolecules* **2018**, *51*, 5519-5529.

[4] H. Yang, N. U. Afsar, Q. Chen, X. Ge, X. Li, L. Ge, T. Xu, *Ind. Chem. Mater.* **2023**, *1*, 129-139.

[5] Y. Li, R. Jia, R. Tan, Z. Hong, J. Gu, P. Cui, S. Zhang, H. Shao, J. Ran, C. F. Fu, *AIChE J.* **2023**, *70*, e18298.

[6] W. Ji, X. Ge, N. U. Afsar, Z. Zhao, B. Wu, W. Song, Y. He, L. Ge, T. Xu, *Sep. Purif. Technol.* **2020**, *247*, 116927.

[7] W. Ji, N. U. Afsar, B. Wu, F. Sheng, M. A. Shehzad, L. Ge, T. Xu, *J. Membr. Sci.* **2019**, *590*, 117267.

[8] R. Mondal, S. Pal, U. Chatterjee, *ACS Appl. Polym. Mater.* **2021**, *3*, 1544-1554.

[9] J. Liu, W. Wu, P. Zuo, Z. Yang, T. Xu, *ACS Macro Lett.* **2024**, *13*, 328-334.

[10] J. Zhang, Y. He, K. Zhang, X. Liang, R. Bance‐Soualhi, Y. Zhu, X. Ge, M. A. Shehzad, W. Yu, Z. Ge, L. Wu, J. R. Varcoe, T. Xu, *AIChE J.* **2021**, *67*, 17133.

[11] P. Ramirez, J. Cervera, S. Nasir, M. Ali, W. Ensinger, S. Mafe, *J. Colloid Interface Sci.* **2024**, *655*, 876-885.

[12] J. Hu, Z. Lv, Y. Xu, X. Zhang, L. Wang, *J. Membr. Sci.* **2016**, *505*, 119-129.

[13] A. Campione, L. Gurreri, M. Ciofalo, G. Micale, A. Tamburini, A. Cipollina, *Desalination* **2018**, *434*, 121-160.

[14] Z. Li, J. Guo, J. Zheng, T. A. Sherazi, S. Li, S. Zhang, *Macromolecules* **2020**, *53*, 10998-11008.

[15] P. Mandal, P. Goel, B. E, V. K. Shahi, S. Chattopadhyay, *J. Environ. Chem. Eng.* **2022**, *10*, 107016.

[16] P. Zuo, C. Ye, Z. Jiao, J. Luo, J. Fang, U. S. Schubert, N. B. McKeown, T. L. Liu, Z. Yang, T. Xu, *Nature* **2023**, *617*, 299-305.

[17] T. Huang, J. Zhang, Y. Pei, X. Liu, J. Xue, H. Jiang, X. Qiu, Y. Yin, H. Wu, Z. Jiang, M. D. Guiver, *Chem. Eng. J.* **2021**, *418*, 129311.

[18] C. Jeon, J. J. Han, M. Seo, *ACS Appl. Mater. Interfaces* **2018**, *10*, 40854-40862.

[19] C. Lin, Y. Gao, N. Li, M. Zhang, J. Luo, Y. Deng, L. Ling, Y. Zhang, F. Cheng, S. Zhang, *Electrochim. Acta* **2020**, *354*, 136693.

[20] P. Zuo, Y. Li, A. Wang, R. Tan, Y. Liu, X. Liang, F. Sheng, G. Tang, L. Ge, L. Wu, Q. Song, N. B. McKeown, Z. Yang, T. Xu, *Angew. Chem. Int. Ed.* **2020**, *59*, 9564-9573.

[21] S. Gong, L. Li, L. Ma, N. A. Qaisrani, J. Liu, G. He, F. Zhang, *J. Membr. Sci.* **2020**, *595*, 117541.

[22] P. Liu, Y. Zheng, S. Lin, Q. Yang, Y. Hong, Q. Zhang, Q. Liu, *J. Power Sources* **2020**, *452*, 227827.

[23] M. Li, M. Sun, W. Liu, X. Zhang, C. Wu, Y. Wu, *Chem. Eng. Res. Des.* **2020**, *153*, 875-886.

[24] Y. Hongxin, L. Xingya, G. Liang, X. J. C. J. Tongwen, *CIESC Journal* **2022**, *73*, 3739-3748.

[25] C. Li, G. Wang, D. Yu, F. Sheng, M. A. Shehzad, T. He, T. Xu, X. Ren, M. Cao, B. Wu, L. Ge, *J. Membr. Sci.* **2019**, *581*, 150-157.

[26] J. Feng, J. Chen, B. Wei, S. Liao, Y. Yu, X. Li, *J. Membr. Sci.* **2019**, *570-571*, 120-129.

[27] J. Sharma, S. Mishra, N. H. Rathod, V. Kulshrestha, *J. Membr. Sci.* **2022**, *664*, 121082.

[28] J. Lin, J. Huang, J. Wang, J. Yu, X. You, X. Lin, B. Van der Bruggen, S. Zhao, *J. Membr. Sci.* **2021**, *624*, 119116.

[29] L. Tian, M. Wang, G. Liao, B. Liu, Y. Sun, Y. Hu, Z. Lu, *Chemistry* **2024**, *30*, e202401361.

[30] A. N. Mondal, C. Cheng, M. I. Khan, M. M. Hossain, K. Emmanuel, L. Ge, B. Wu, Y. He, J. Ran, X. Ge, N. U. Afsar, L. Wu, T. Xu, *J. Membr. Sci.* **2017**, *525*, 163-174.

[31] M. Irfan, N. U. Afsar, Y. Wang, T. Xu, *J. Taiwan Inst. Chem. Eng.* **2018**, *93*, 405-413.

[32] N. U. Afsar, B. Erigene, M. Irfan, B. Wu, T. Xu, W. Ji, K. Emmanuel, L. Ge, T. Xu, *Sep. Purif. Technol.* **2018**, *193*, 11-20.

[33] J. Ran, M. Hu, D. Yu, Y. He, M. A. Shehzad, L. Wu, T. Xu, *J. Membr. Sci.* **2016**, *520*, 630-638.

[34] X. H. Li, N. Ul Afsar, X. P. Chen, Y. F. Wu, Y. Chen, F. Shao, J. X. Song, S. Yao, R. Xia, J. S. Qian, B. Wu, J. B. Miao, *Membranes* **2022**, *12*, 12.

[35] T. Deng, X. J. Zeng, C. Y. Zhang, Y. X. Wang, W. Zhang, *Chem. Eng. J.* **2022**, *445*, 8.

[36] C. Yang, L. Hou, Z. Yao, J. Zhao, L. a. Hou, L. Zhang, *J. Membr. Sci.* **2021**, *640*, 119800.
